# Rapamycin immunomodulation utilizes time-dependent alterations of lymph node architecture, leukocyte trafficking, and gut microbiome

**DOI:** 10.1172/jci.insight.186505

**Published:** 2025-04-22

**Authors:** Long Wu, Allison Kensiski, Samuel J. Gavzy, Hnin Wai Lwin, Yang Song, Michael T. France, Ram Lakhan, Dejun Kong, Lushen Li, Vikas Saxena, Wenji Piao, Marina W. Shirkey, Valeria R. Mas, Bing Ma, Jonathan S. Bromberg

**Affiliations:** 1 Department of Surgery,; 2 Center for Vascular and Inflammatory Diseases,; 3 Institute for Genome Sciences, and; 4 Department of Microbiology and Immunology, University of Maryland School of Medicine, Baltimore, Maryland, USA.

**Keywords:** Immunology, Microbiology, Laminin, T cells, Tolerance

## Abstract

Transplant recipients require lifelong, multimodal immunosuppression to prevent rejection by reducing alloreactive immunity. Rapamycin is known to modulate adaptive and innate immunity, but its full mechanism remains incompletely understood. We investigated the understudied effects of rapamycin on lymph node (LN) architecture, leukocyte trafficking, and gut microbiome and metabolism after 3 (early), 7 (intermediate), and 30 (late) days of rapamycin treatment. Rapamycin significantly reduced CD4^+^ T cells, CD8^+^ T cells, and Tregs in peripheral LNs, mesenteric LNs, and spleen. Rapamycin induced early proinflammation transition to protolerogenic status by modulating the LN laminin α4/α5 expression ratios (La4/La5) through LN stromal cells, laminin α5 expression, and adjustment of Treg numbers and distribution. Additionally, rapamycin shifted the *Bacteroides*/*Firmicutes* ratio and increased amino acid bioavailability in the gut lumen. These effects were evident by 7 days and became most pronounced by 30 days in naive mice, with changes as early as 3 days in allogeneic splenocyte-stimulated mice. These findings reveal what we believe to be a novel mechanism of rapamycin action through time-dependent modulation of LN architecture and gut microbiome, which orchestrates changes in immune cell trafficking, providing a framework for understanding and optimizing immunosuppressive therapies.

## Abstract 2

Rapamycin exerts time-dependent effects on both the immune system and gut microbiome, transitioning from initial pro-inflammatory responses to later pro-tolerogenic states, with significant alterations in lymph node architecture, T cell trafficking, and microbial metabolism, revealing novel mechanisms beyond mTOR inhibition and suggesting potential for optimized, temporal immunosuppression strategies.

## Introduction

Prevention of solid organ transplant rejection requires the use of lifelong, multimodal immunosuppression to dampen both adaptive and innate alloreactive immunity. One major mainstay is rapamycin (sirolimus), a bacterial fermentation product recognized for its immunosuppressive and antiproliferative properties through the inhibition of the mechanistic target of rapamycin (mTOR), a critical kinase in cell cycle regulation and immune response modulation (1, 2). The mTOR pathway is a key regulator of cellular metabolism and growth, influencing nutrient availability, growth factors, and stress responses. Rapamycin has been shown to promote autophagy, delay cellular senescence, and act as an antiproliferative agent in certain cancers (3, 4). The antiproliferative effects are beneficial in calcineurin inhibitor–free regimens, preserving renal function and reducing the incidence of posttransplant cancers (5), although these same properties can lead to side effects such as increased infection risk, impaired wound healing, and metabolic disturbances (6). Despite its established role in mTOR inhibition, the effect of rapamycin on other immune populations and its precise mode of action has not been fully investigated. Given its broad effect on immune landscape, it is critical to understand its molecular mechanisms for optimizing its clinical use and enhancing longevity in patients who have had solid organ transplants.

Rapamycin targets mTOR complex 1 (mTORC1) and mTORC2 and blocks the activation and proliferation of T and B cells in response to cytokine stimulation to exert immunosuppressive effects (3, 4, 7). Rapamycin modulates various signaling pathways, promoting the maintenance of memory T cells and improving their ability to respond to subsequent exposures to the same antigen (8, 9). It also preserves and supports the maturation-resistance and tolerogenic properties of DCs. Rapamycin modulates innate immunity by promoting an antiinflammatory phenotype in DCs via enhanced mitochondrial metabolism and increased IL-10 production (7). Recent studies demonstrate its ability to delay the onset of cellular senescence and regulate metabolic pathways similar to caloric restriction, interventions known to extend lifespan in various species (3). Rapamycin regulation of autophagy, a process required for maintaining amino acid levels and protein synthesis during nitrogen starvation, can influence amino acid balance and related cellular metabolism and immune responses (10).

Beyond their primary action on immune cell populations, immunosuppressants can also affect the gut microbiome, thus influencing alloimmunity and graft survival (11–15). In this way, immunosuppressants elicit secondary effects on both local and systemic immune responses, which can then influence allograft outcomes (16–20). Recent work revealed a complex interplay between the commensal gut microbiome, immunosuppressant therapies, and the host immune system (17, 21–24), highlighting that immunosuppressant-induced changes in the gut microbiome, alongside the use of antimicrobials, can affect overall immune homeostasis. Immunosuppressants primarily affect anaerobic bacteria, including *Ruminococcaceae*, *Lachnospiraceae*, *Firmicutes*, *Bacteroides*, and *Clostridiales* (25–27). Furthermore, immunosuppressants use has been associated with an increase in colonization of uropathogenic *E. coli* and *Enterococcus faecium* (20, 28, 29). Overall, the effect of immunosuppressants extends beyond direct effects on immune cells to include modulation of the gut microbiome. However, the precise alterations in microbiome composition and function induced by rapamycin, along with their underlying mechanisms, remain to be elucidated.

In addition to multidirectional gut microbiome–endothelium–immune cell interactions, rapamycin’s effect on other lymphoid organs, particularly lymph nodes (LNs), remains poorly understood. LN structure relies on LN stromal cells (LNSCs) to regulate the position and interaction of lymphocytes with antigen-presenting cells (APCs) via chemokines, cytokines, and stromal fibers (30–32). LNSCs, which include fibroblastic reticular cells (FRCs), lymphatic endothelial cells (LECs), and blood endothelial cells (BECs), form a critical infrastructure that supports immune cell trafficking and interactions within the LN microenvironment. FRC-derived laminins play a crucial role in modulating global immune states by balancing tolerance and immunity (33). Laminins, as extracellular matrix proteins, influence the structural integrity of the LN and affect immune cell behavior (33). Laminin α4 is associated with a tolerogenic niche, while laminin α5 is linked to immunity and inflammation. An increased La4/La5 correlates with tolerance, whereas a decreased ratio is associated with inflammation and immunity (34, 35). For instance, depleting laminin α5 in LNSCs has been shown to promote a tolerogenic environment, increasing Treg migration and accumulation in specific LN regions, thus contributing to immunosuppression (33, 36). Changes in LN architecture driven by laminins can be induced by various factors, including the gut microbiota and alloimmunity (23). For example, the gut microbiota can influence LN structure through microbial metabolites and immune signaling (23). Similarly, alloimmunity can lead to structural changes in the LN, affecting immune tolerance and graft survival (37). Additionally, ischemia reperfusion injury of the graft during transplantation can disrupt LN structure and FRC function, leading to immunologic scarring (38, 39). Pathologic alterations to this cellular network pose challenges for maintaining immune homeostasis and preventing chronic graft rejection. Investigating rapamycin effects on LN architecture and LNSCs offers key insights into its immunosuppression mechanisms.

Treg positioning within the LNs is a pivotal factor in determining the balance between proinflammatory and protolerogenic states in maintaining immune homeostasis. Tregs around the high endothelial venules (HEV) limit the entry of proinflammatory effector T cells into the LNs (33), helping maintain a tolerogenic environment by preventing excessive accumulation of proinflammatory cells. Tregs in the cortical ridge (CR) suppress T cell priming by APCs, preventing effector T cell activation and differentiation (40). A reduction of Tregs in the CR can result in enhanced T cell activation, promoting a proinflammatory state (37, 41, 42). This spatial organization of immune regulation underscores the importance of maintaining proper Treg positioning for effective immunosuppression and tolerance induction.

This study investigates the contribution of rapamycin to LN architecture, leukocyte trafficking, gut microbiome, and the mechanisms underlying these changes. We examined microanatomic cell positioning and interactions, as well as LN stromal fiber structure, building on our previous work that demonstrated the importance of architectural and cellular changes within the LN cortex, including the CR and HEVs, in mediating immune tolerance and suppression (33, 36). We performed temporal characterization after 3 (early), 7 (intermediate), and 30 (late) days of treatment. We revealed dynamic, time-dependent effects of rapamycin on LN architecture and Treg localization, showing a transition from early proinflammatory changes to a later protolerogenic environment. This transition was mediated by the modulation of the La4/La5 in LNSCs via altering laminin α5 expression, with a higher La4/La5 indicative of a tolerogenic environment. These observations were validated across naive, laminin α4 KO, laminin α5 KO, and allogeneic splenocyte-stimulated mice. Furthermore, rapamycin significantly altered gut microbiota composition, intestinal Tregs, and metabolic functions. These effects were evident by 7 days but most pronounced by 30 days, emphasizing the incremental and dynamic effect of rapamycin. Our study reveals multifaceted, time-dependent rapamycin effects on immune regulation via LN architecture and gut microbiome, offering what we believe to be a new lens for optimizing long-term immunosuppressive therapies to enhance graft survival and patient longevity.

## Results

### Rapamycin reduces lymphocytes in LNs and spleen.

**Figure 1:**
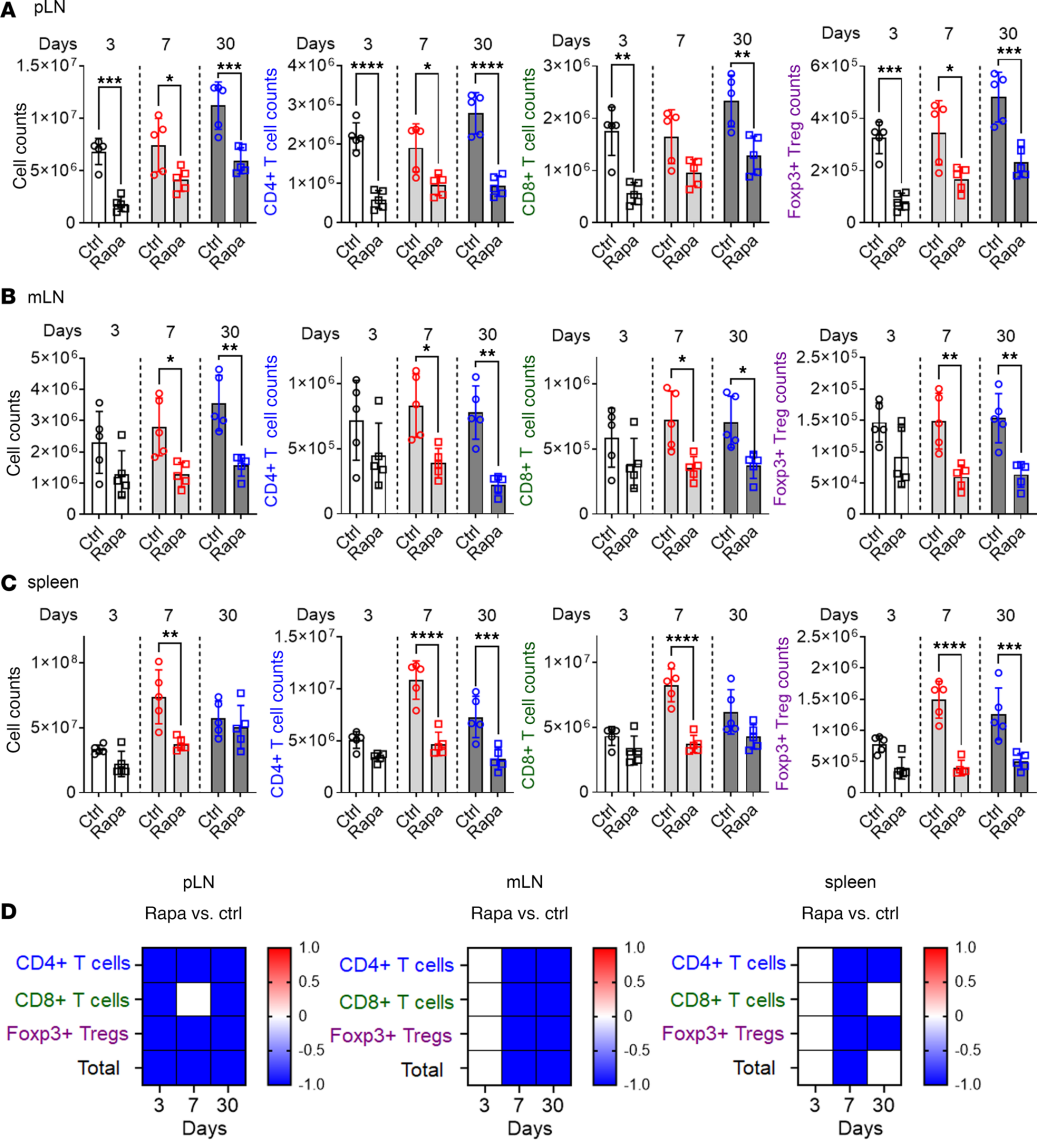
Rapamycin elicits significant changes in immune cell populations in LNs and spleen. (**A**–**C**) Flow cytometry for the total number (CD45^+^ cells), CD4^+^ T cells, CD8^+^ T cells, and Foxp3^+^ Tregs (Foxp3^+^CD4^+^) in pLN (**A**), mLN (**B**), and spleen (**C**) after 3, 7, and 30 days of rapamycin treatment. (**D**) Heatmap depicts changes in cell numbers relative to the control for pLN, mLN, and spleen after rapamycin treatment versus no drug control; red represents “increased,” white represents “unchanged,” and blue represents “decreased.” There were 5 mice/group. One-way ANOVA. *P < 0.05, **P < 0.01, ***P < 0.001, ****P < 0.0001.

To investigate the immunological effects of rapamycin within secondary lymphoid organs, C57BL/6 mice received rapamycin (5 mg/kg/day i.p.) (43) and were characterized after 3 (early), 7 (intermediate), and 30 (late) days of treatment. We assessed cell populations in peripheral LNs (pLNs), mesenteric LNs (mLNs), and spleen, focusing on CD4^+^ and CD8^+^ T lymphocytes and Tregs via flow cytometry. Reductions in pLN weight and overall cell counts were observed at all 3 time points, consistent with known rapamycin inhibitory effects on cell proliferation and metabolism (Supplemental Figure 1, A–D; supplemental material available online with this article; https://doi.org/10.1172/jci.insight.186505DS1). Rapamycin reduced CD4^+^ and CD8^+^ T lymphocytes and Foxp3^+^ Tregs at all time points in the pLNs (Figure 1, A and D), demonstrating substantial and sustained immunomodulatory effects. In the mLNs, rapamycin reduced CD4^+^ and CD8^+^ T lymphocytes and Foxp3^+^ Tregs after 7 and 30 days but not on day 3 (Figure 1, B and D). In the spleen, decreases in CD4^+^ T cells and Foxp3^+^ Tregs were noted on days 7 and 30, with decreased CD8^+^ T cells only on day 7 (Figure 1, C and D). Rapamycin decreased the Treg/non-Treg ratio in the spleen but not in LNs (Supplemental Figure 1, E–G), suggesting an uneven effect on various T cell populations. Overall, these data demonstrate that rapamycin markedly reduced lymphocytes and Treg cell counts in LNs and spleen, and this is a manifestation of its immunosuppressive effect. Notably, rapamycin showed more pronounced effects in mLNs and spleen at later time points, while earlier changes were primarily observed in pLNs. This suggests that rapamycin immunomodulatory effects are both time dependent and site specific.

### Time-dependent effect of rapamycin on LN architecture and Treg distribution.

**Figure 2:**
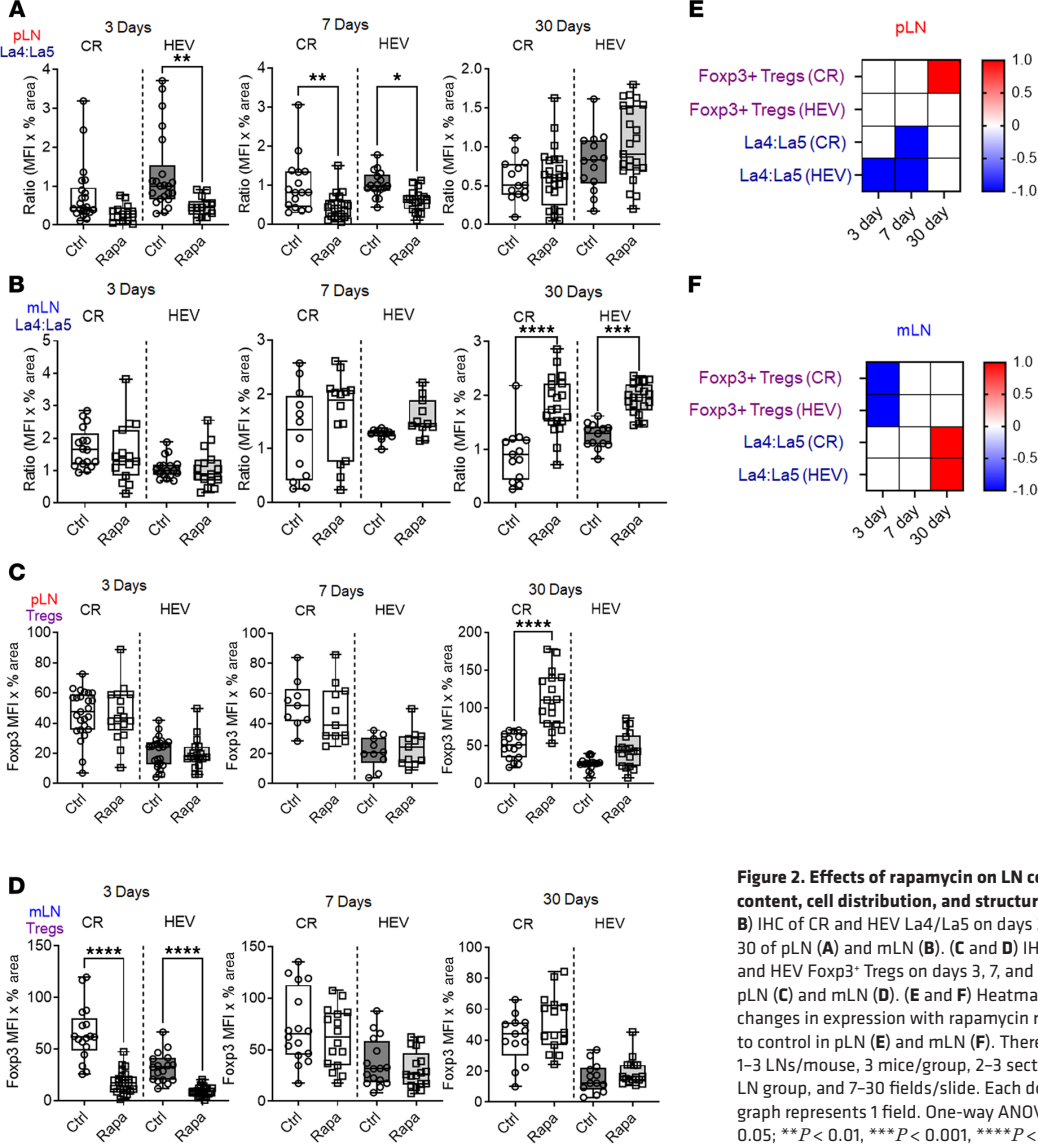
Effects of rapamycin on LN cell content, cell distribution, and structure. (**A** and **B**) IHC of CR and HEV La4/La5 on days 3, 7, and 30 of pLN (**A**) and mLN (**B**). (**C** and **D**) IHC of CR and HEV Foxp3^+^ Tregs on days 3, 7, and 30 of pLN (**C**) and mLN (**D**). (**E** and **F**) Heatmaps depict changes in expression with rapamycin relative to control in pLN (**E**) and mLN (**F**). There were 1–3 LNs/mouse, 3 mice/group, 2–3 sections/LN group, and 7–30 fields/slide. Each dot in the graph represents 1 field. One-way ANOVA. *P < 0.05; **P < 0.01, ***P < 0.001, ****P < 0.0001.

The architecture and distribution of specific immune cells within the LN microenvironments, including the CR and HEVs, are key in fostering immune tolerance and suppression (37). We conducted quantitative IHC of these LN domains to characterize rapamycin spatiotemporal effects. Rapamycin rapidly induced early proinflammatory changes by decreasing the La4/La5 in pLN HEVs by day 3. This extended to pLN CR by day 7 and diminished to undetectable levels by day 30 (Figure 2, A and E, and Supplemental Figure 2, A and B), indicating an early proinflammatory effect. In mLNs, rapamycin increased La4/La5 on day 30, with no changes on days 3 or 7 (Figure 2, B and F, and Supplemental Figure 2C), indicating a late protolerogenic effect.

Using IHC, in pLNs, rapamycin increased Foxp3^+^ Tregs by day 30, especially in the CR, without alterations at earlier time points (Figure 2, C and E, and Supplemental Figure 2D). Flow cytometry analysis of total pLN Treg percentages corroborated these findings, demonstrating stable levels through days 3 and 7, followed by an increase at day 30 (Supplemental Figure 1H), indicating a late protolerogenic effect. In mLNs, rapamycin reduced Foxp3^+^ Tregs on day 3 but not at later time points (Figure 2, D and F, and Supplemental Figure 2E). Analysis of total mLN Foxp3^+^ Treg percentages revealed an increase by day 30 (Supplemental Figure 1I), suggesting a temporal transition in rapamycin effects from early localized proinflammatory to later protolerogenic states. Overall, both pLNs and mLNs exhibited early proinflammatory states, characterized by decreased La4/La5 or reduced Tregs followed by a late protolerogenic environment, marked by either increased La4/La5 or elevated Tregs. Notably, the data indicate that day 7 marked a transition from proinflammatory to protolerogenic regulation by rapamycin.

### Rapamycin increases laminin α5 and decreases La4/La5 in LNSCs.

**Figure 3:**
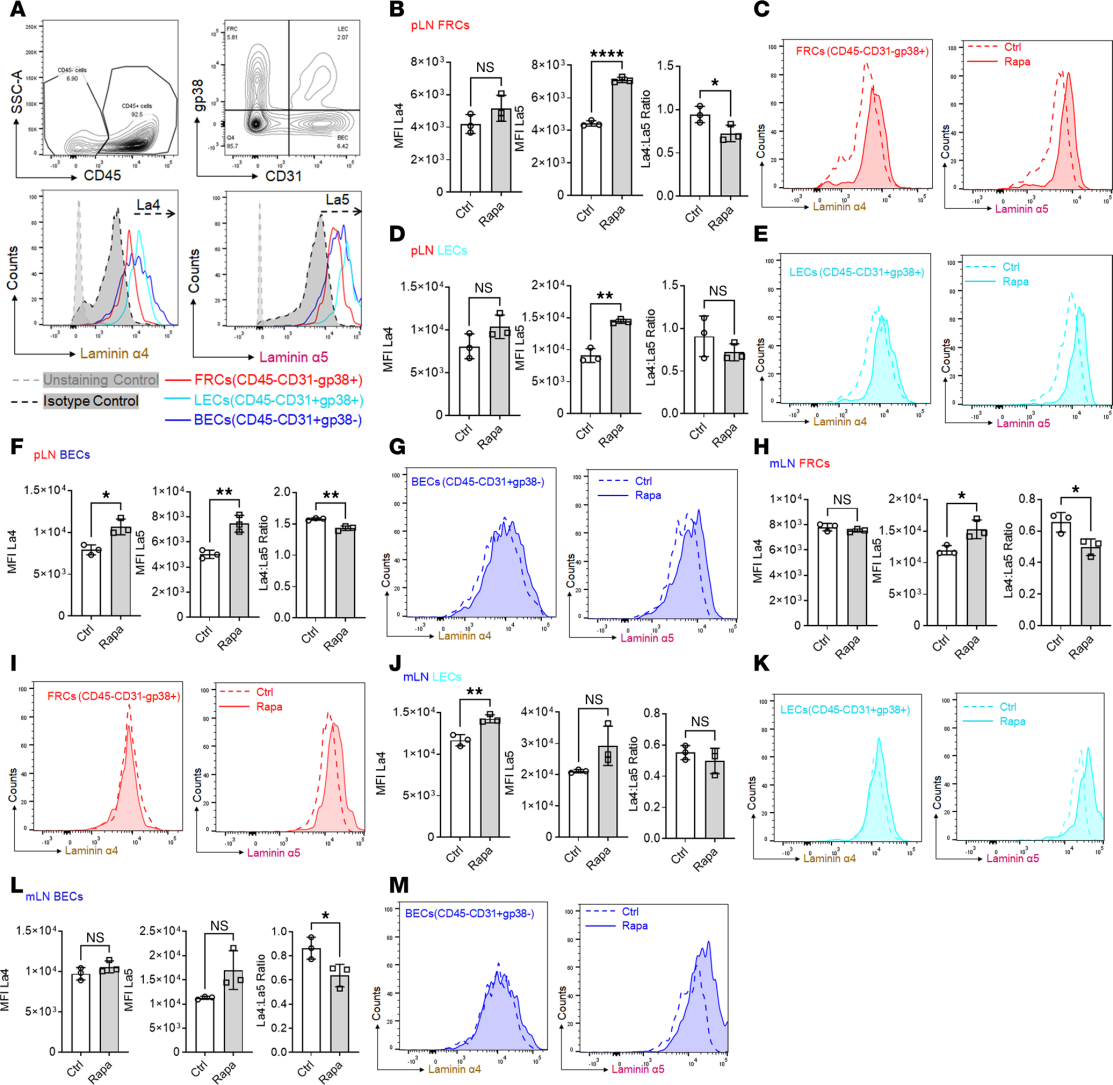
Rapamycin increases laminin α5 and decreases the La4/La5 in LNSCs. (**A**) Flow gating of CD45^–^ cells for FRCs (CD31^–^gp38^+^), BECs (CD31^+^gp38^–^), and LECs (CD31^+^gp38^+^) for laminin α4 (La4) and laminin α5 (La5). (**B**–**M**) Mean fluorescence intensity (MFI) and flow plots show La4, La5, and La4/La5 ratios in: pLN (**B**–**G**), FRCs (**B** and **C**), LECs (**D** and **E**), BECs (**F** and **G**); mLN (**H**–**M**); FRCs (**H** and **I**); LECs (**J** and **K**); and BECs (**L** and **M**). There were 3 mice/group. Two-tailed *t* test. *P < 0.05, **P < 0.01, ****P < 0.0001.

Given the effect of rapamycin on LN architecture via La4/La5, we next sought to determine whether LNSC expression of laminin α4, laminin α5, or both directly mediated these effects after 7 days treatment, given that this time point marked the critical transition from proinflammation to protolerance. Flow cytometry was used to quantify laminin α4 and α5 levels in live CD45^–^ LNSCs, including FRCs, BECs, and LECs. In pLNs, rapamycin upregulated laminin α5 in all groups with little influence on laminin α4, leading to decreased La4/La5 (Figure 3, A–G). In mLNs, treatment with rapamycin similarly decreased the La4/La5 in LNSCs by upregulating laminin α5 expression (Figure 3, H–M). Taken together, this cell type–specific analysis provided detailed insight into how rapamycin modulates proinflammatory responses by selectively increasing laminin α5 expression in LNSCs, hence decreasing La4/La5. The flow cytometry analysis revealed a subset-specific increase in laminin α5 expression within LNSCs (Figure 3), with no notable changes in laminin α4 levels. By contrast, IHC provided a broader architectural analysis of La4/La5 across all cell types and in specific regions (Figure 2). This broader approach, which does not differentiate between cell types, reflects contributions from both preexisting laminin expression along with laminin expression from nonstromal cells, diluting the changes in laminin α5 observed in stromal subsets.

### Laminin α5 is responsible for rapamycin-induced changes in LN architecture.

**Figure 4:**
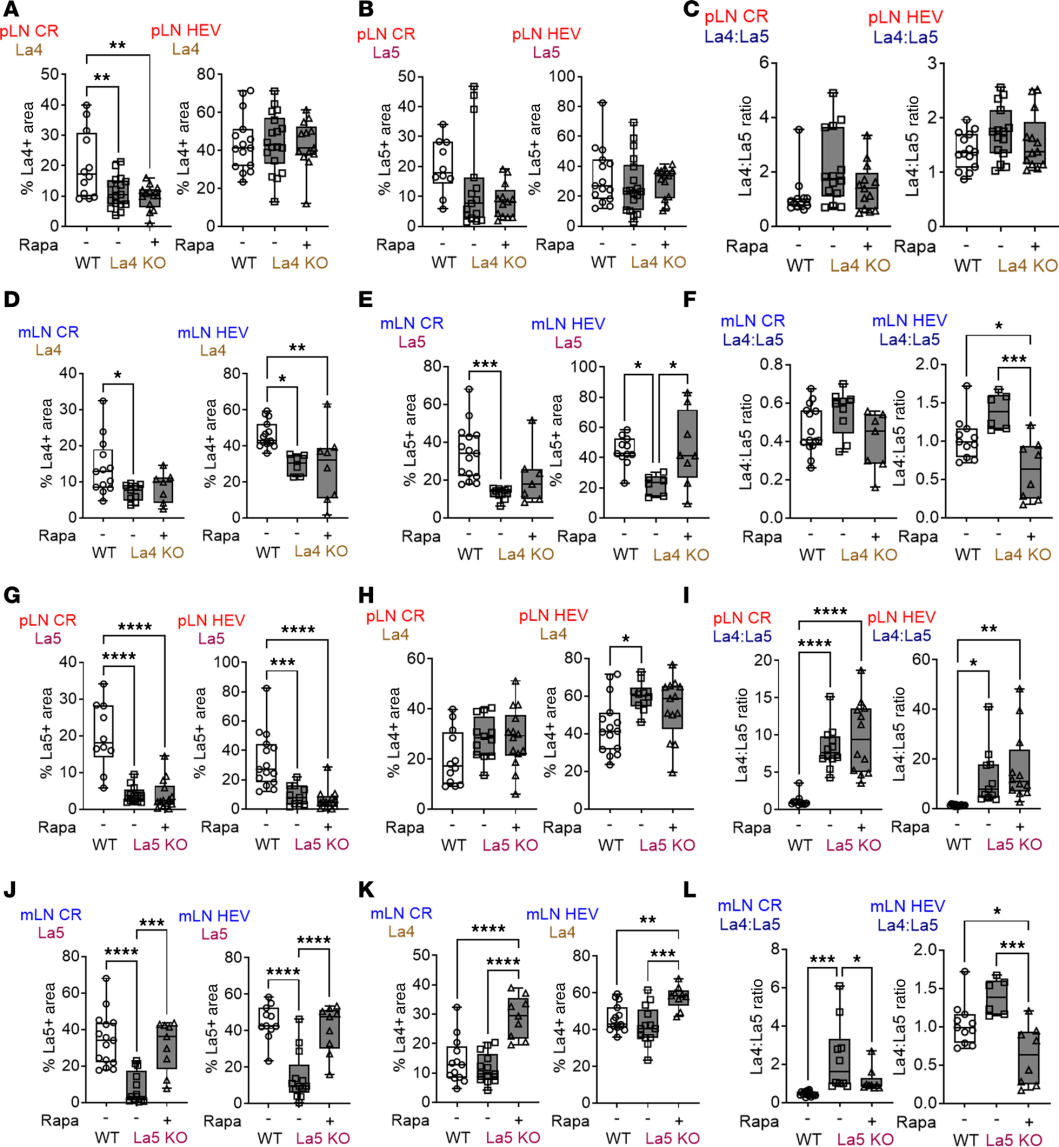
Rapamycin regulates laminin α5 in FRC-La4-KO and FRC-La5-KO mice. IHC showing (**A** and **H**) laminin α4; (**B** and **G**) laminin α5; (**C** and **I**) La4/La5 in pLN; (**D** and **K**) laminin α4; (**E** and **J**) laminin α5; (**F** and **L**) La4/La5 in mLN for WT, untreated, and rapamycin-treated FRC-La4-KO mice (**A**–**F**) and FRC-La5-KO mice (**G**–**L**) on day 3. There were 1–3 LNs/mouse, 3–5 mice/group, 2–3 sections/LN group, and 7–30 fields/slide. Each dot in the graph represents one field. One-way ANOVA. *P < 0.05, **P < 0.01, ***P < 0.001, ****P < 0.0001.

Given the effect of rapamycin on pLNs La4/La5 on day 7 and the important role of FRCs in laminin expression, we further employed 2 laminin-KO strains to assess if laminin α4 or α5 expression was mediated by FRCs. FRC–laminin α4–KO (FRC-La4-KO) mice (Pdgfrb-Cre^+/–^ × La4^fl/fl^) have the laminin α4 gene deleted in FRCs. At baseline, these mice showed decreased laminin α4 levels in both pLNs and mLNs compared with WT mice (Figure 4, A and D). Administration of rapamycin did not affect the expressions of laminin α4, laminin α5, and the La4/La5 in pLNs of the FRC-La4-KO mice (Figure 4, A–C, and Supplemental Figure 3A). In mLNs, rapamycin increased laminin α5 around HEV without affecting laminin α4 in FRC-La4-KO, leading to a reduced La4/La5 (Figure 4, E and F, and Supplemental Figure 3B). These results indicate that, even in the absence of FRC laminin α4, rapamycin can still upregulate laminin α5 expression, suggesting that FRC-derived laminin α4 is not required to mediate the LN architectural change under the influence of rapamycin.

FRC-La5-KO mice (Pdgfrb-Cre^+/–^ × La5^fl/fl^) have the laminin α5 gene deleted in FRCs. At baseline, these mice showed decreased laminin α5 in both pLN and mLN compared with WT mice (Figure 4, G and J). Administration of rapamycin did not affect the expression of laminin α4 or α5 in the pLN, resulting in an unchanged La4/La5 in FRC-La5-KO mice (Figure 4, G–I, and Supplemental Figure 3A). In the mLN, there was a differential increase in both laminin α4 and α5 (Figure 4, J and K), leading to a decrease in the La4/La5 (Figure 4L and Supplemental Figure 3B). Since the laminin α5 gene is deleted in FRCs but not in other cells, the increased laminin α5 levels after rapamycin treatment suggest upregulation of laminin α5 expression by non-FRC cell types, such as LECs and BECs. Flow cytometry results (Figure 3) support this conclusion. Overall, the results show that rapamycin differentially regulates laminin α4 and α5, altering LN architecture. This effect is not exclusively mediated by FRC-derived laminin, as LECs and BECs also play crucial roles in these rapamycin-induced changes.

### Time-dependent effect of rapamycin on gut microbiota composition and metabolic capacity.

**Figure 5:**
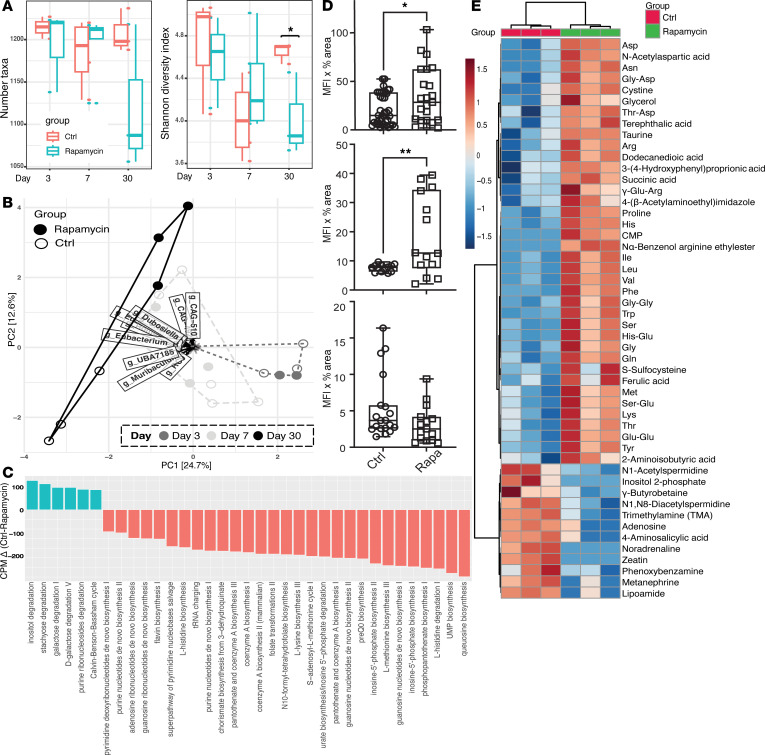
Rapamycin alters gut microbiome and metabolic potentials. (**A**–**C**) Gut microbiome characterization of rapamycin treated and no-treatment control for within-community diversity by total number of taxa and Shannon diversity index (**A**); community β-diversity with PCA (**B**); and gut microbiome functional pathway abundance in copies per million (CPM) difference between control and rapamycin groups (**C**). The height of the stacked bar represents CPM of associated MetaCyc pathways contributed by different taxa in control (green) or rapamycin (red). There were 3–5 mice/group and 1 stool sample collected/mouse/time point. (**D**) Intestinal IHC of Foxp3^+^ Tregs after 3 days, 7 days, and 30 days of rapamycin treatment. There were 3 mice/group as a block, 1 piece of intestine, 2–3 sections/block on a slide, and 7–30 fields/slide. Each dot in the graph represents 1 field of view from the slide. One-way ANOVA. *P < 0.05, **P < 0.01. (**E**) Hierarchical clustering heatmap of metabolites of rapamycin and control groups. Specimens were collected after rapamycin treatment for 7 days, compared with no drug control. Top 50 features shown. Color bar indicates the scaled *z* score of each feature.

We next investigated rapamycin effects on the gut microbiome because of its critical role in interfacing with the immune system. Whole-community metagenomic sequencing of intraluminal fecal contents was performed at a sequencing depth of 41.1 ± 14.5 (mean ± SD) million reads after quality control steps per sample (Supplemental Table 2A). Taxonomic composition was estimated using the comprehensive mouse microbiota genome catalog (44) (Supplemental Table 2B). After rapamycin treatment, no significant differences in gut microbiota diversity were observed at days 3 and 7 (Figure 5A). However, by day 30, there was a decrease in community diversity (*P* < 0.05) and a shift in microbial composition and structure, characterized by increased *Bacteroides* to *Firmicutes* relative abundance ratio (B/F ratio) (Supplemental Figure 4A). The B/F ratio on day 3 was 0.29 ± 0.26 (mean ± SD), increased to 0.78 ± 0.53 on day 7, and reached 1.19 ± 1.13 by day 30, indicating a sustained effect of rapamycin on the structure and composition of the gut microbiota. Compared with the control group, no significant changes in taxonomic groups were observed on day 3. In *Bacteroides*, *Duncaniella* showed the most pronounced increase at day 7 and *Muribaculum* was most increased at day 30, in response to rapamycin treatment (Supplemental Figure 4B). In *Firmicutes*, *Lachnospiraceae*, *Lactobacillales*, and *Christensenellales* showed the greatest decrease under rapamycin treatment at 30 days (Supplemental Figure 4C). Using differential abundance analyses, sporadic alterations were noted on day 7 including *Bacteroidale* (i.e., *Duncaniella sp.*, *Muricubaculum intestinale*) and *Firmicutes* (i.e., *Eubacterium*) (Supplemental Figure 4D and Supplemental Table 2C). By day 30, there was an increase in *Bacteroidales* (including *Muribaculaceae, Bacteroides, Actinobacteria*), and a decrease in *Firmicutes* (such as *Lachnospiraceae*, *Oscillibacter*, *Lawsonibacter*, *Eubacterium*) (Supplemental Figure 4E). These taxonomic groups drive the observed changes in composition and structure of the gut microbiota at different times and between groups (Figure 5B). These effects, starting at day 7 and most pronounced by day 30, highlight a sustained and potent influence of rapamycin on the microbiome and correspond to the same time frame for changes in LN immune architecture and content.

Functional pathway characterization revealed an increase in nucleotide biosynthesis and a reduction in glycolysis and multiple sugar degradation metabolic pathways after 30 days of rapamycin treatment (Figure 5C) but not 3 or 7 days. Species-resolved functional pathway analysis revealed that the *Muribaculaceae* family (Bacteroides) was enriched after long-term rapamycin treatment and harbored pathways involved in nucleotide biosynthesis (Supplemental Figure 5A). However, *B*. *thetaiotaomicron* and a variety of Clostridiales taxa (Firmicutes) that were relatively depleted after long-term rapamycin treatment harbored functional pathways in amino acid biosynthesis (i.e., ornithine), branched and aromatic amino acid biosynthesis (horismate pathway), glycolysis, and energy processing (Supplemental Figure 5B). These findings revealed a distinct shift in the gut microbiome composition and function following rapamycin treatment, emphasizing rapamycin effects beyond immunosuppression and a potential mechanism for its diverse therapeutic effect.

### Rapamycin temporally shifts intestinal immune responses.

To elucidate the reciprocal interactions between the gut microbiome and host under rapamycin treatment, we analyzed the intestinal transcriptome. Days 7 and 30 were chosen as they corresponded to the major gut microbiota alterations following rapamycin treatment. Differentially expressed genes (DEGs) were identified by comparing the rapamycin group to the no-treatment control. A total of 69 and 234 upregulated DEGs were observed at days 7 and 30, respectively, and a total of 168 and 84 downregulated DEGs were observed at days 7 and 30, respectively (Supplemental Table 3, A and B). At day 7, 70.9% of DEGs were downregulated, while at day 30, 73.6% of DEGs were upregulated. Analysis of differential expression patterns, visualized using an UpSet plot, revealed distinct temporal responses to rapamycin treatment with minimal overlap between day 7 and day 30 (Supplemental Figure 6A). Of all DEGs, only 32 genes (16 upregulated and 16 downregulated) showed consistent modulation at both time points. Many changes were time point specific: 159 genes were uniquely regulated at day 7 (45 upregulated and 114 downregulated), while 121 genes were uniquely modulated at day 30 (82 upregulated and 39 downregulated). The day 7 response primarily reflected suppression of inflammatory and innate immune pathways as well as chromatin remodeling (Supplemental Table 3C). Downregulated genes included multiple immunoglobulin families (Ighv, Igkv), immune defense genes (GTPases Igtp, Iigp1, Irgm1, Irgm2), antimicrobial peptides (Reg3b, Reg3g, Defa family), and inflammatory mediators (Ccl8, Ccl24, Cxcl9). Upregulated genes included histone family members (H2bc, H4c), metabolic regulators (Scd2, Tmprss15), and immune modulators (Nos2, Ubd). By day 30, the response shifted toward metabolic reprogramming and selective immune modulation. Downregulated genes were involved in glucose metabolism (G6pc, Pck1), lipid metabolism (Srebf1, Cyp4a10), and stress response. Upregulated genes included B cell–related genes (Cd19, Cd79a, Blk, Ms4a1), MHC class II pathway components (H2-Aa, Ciita), metabolic modulators (Cyp2c55, Slc10a2), and defense peptides (Defa family). The small set of consistently regulated genes across both time points maintained aspects of B cell regulation and antimicrobial defense. Consistently downregulated genes included immunoglobulin family members (Ighv) and metabolic regulators, while consistently upregulated genes included B cell–related genes, antimicrobial peptides (Defa family), and metabolic regulators. This temporal pattern suggests that rapamycin induces distinct phases of intestinal adaptation, transitioning from broad immunosuppression at day 7 to more targeted metabolic and immune regulatory programs by day 30.

On the other hand, intestinal Foxp3^+^ Treg expression was strongest at days 3 and 7 but was attenuated by day 30 (Figure 5D and Supplemental Figure 7). The enriched upregulated immune pathways at both days 7 and 30 include B cell regulation, activation, proliferation, antigen binding, and immunoglobulin-mediated immune responses (Supplemental Figure 6B). On day 7, there was unique enrichment in cellular responses to interferon-λ, -α, and -β, as well as cytokine-mediated signaling pathways. At day 30, unique enrichment was observed in MHC class II protein complex binding, antigen processing and presentation, mucosal immune responses, and tissue-specific immune responses. Other immune pathways demonstrated a substantially stronger effect in most functional categories by day 30, such as immunoglobulin receptor binding, production, and circulation; positive regulation of lymphocyte activation; and phagocytosis (Supplemental Figure 6C). Overall, the transcriptional changes were substantial, marked by the number of DEGs and enriched pathways. These results collectively indicate a temporal shift from suppression to activation in both intestinal gene expression and the immune environment following rapamycin treatment.

### Rapamycin reprograms amino acid metabolism in gut lumen.

Given the alterations in the composition and functional makeup of the gut microbiome, we next assessed whether this translated to functional changes in metabolism through the gut luminal metabolome. Intraluminal stool was assessed using capillary electrophoresis–mass spectrometry (CE/MS) (45–47). The 7-day time point was used as it represents the transitional phase between early and late alloimmune responses in both LNs and the intestine. A no-treatment group served as control to provide a baseline for comparison. Luminal metabolites (*n* = 264) were exhaustively annotated by PubChem (48), Kyoto Encyclopedia of Genes and Genomes (KEGG) (49), and Human Metabolome Database (HMDB) (50) (Supplemental Table 4A). According to the KEGG BRITE hierarchical classification system, the most prevalent class of luminal metabolites was from amino acid metabolism, comprising 42.7% of all annotated metabolites (Supplemental Table 4B). These metabolites belonged to pathways of arginine and proline, histidine, tyrosine, and tryptophan metabolism. Other prevalent classes included carbohydrates (11.5%), cofactors and vitamins (10.4%), nucleotides (10.4%), lipids (6.3%), other amino acids (6.3%), and xenobiotic metabolism (5.2%). Distinct gut metabolic profiles were observed after rapamycin treatment, with amino acids such as Asn, Phe, Arg, and Leu and metabolic derivatives differentially abundant in rapamycin treatment group (Figure 5E and Supplemental Figure 8, A and B). These results suggest that rapamycin treatment either increased amino acid biosynthesis and/or reduced catabolism.

### Rapamycin induces a rapid proinflammatory response and a gut microbiome shift during allogeneic stimulation.

**Figure 6:**
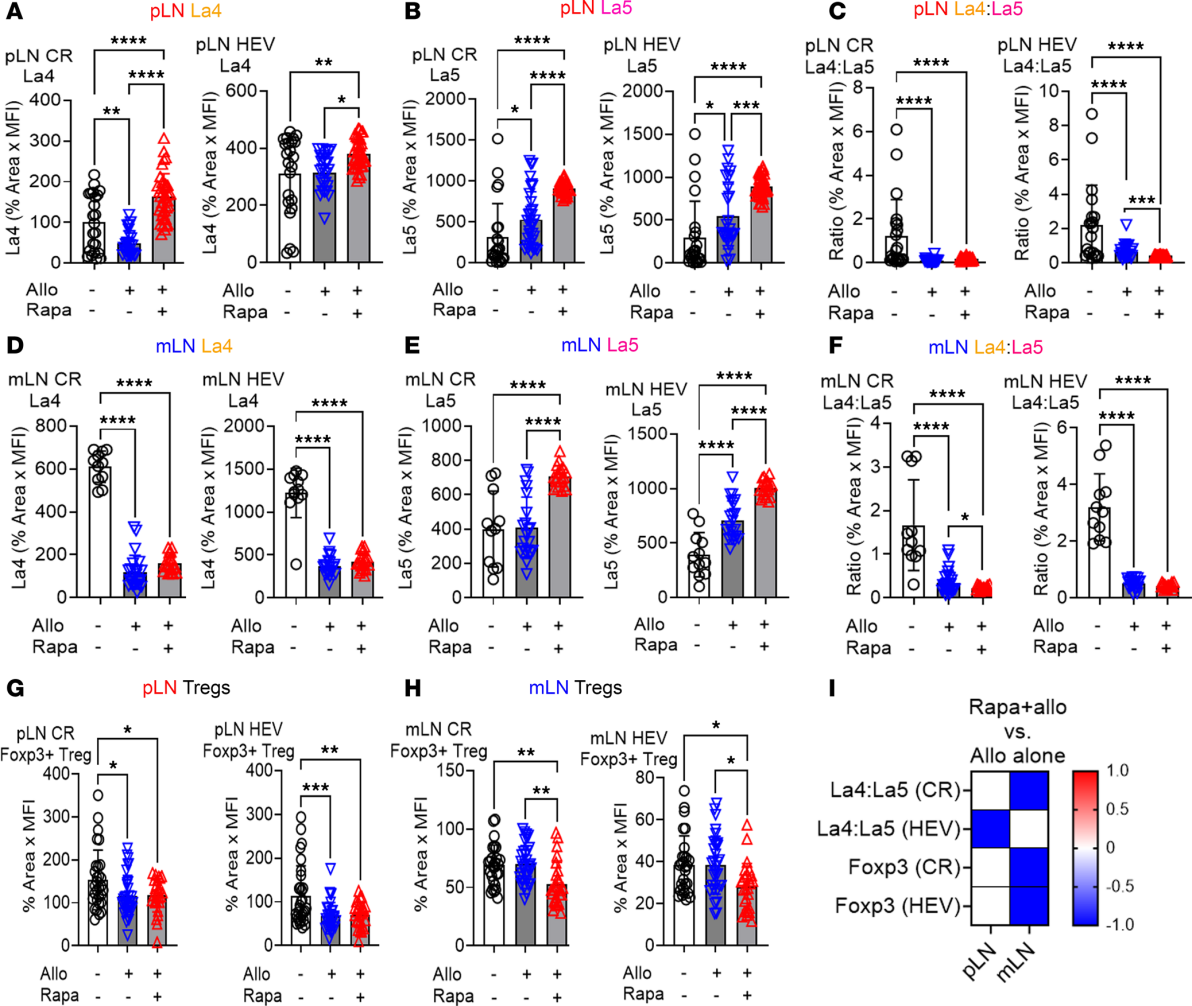
Rapamycin triggers a rapid inflammatory immune response in mice immunized with allogeneic splenocytes. (**A**–**C**) IHC for pLN laminin α4 (**A**), laminin α5 (**B**), and La4/La5 (**C**). (**D**–**F**) IHC for mLN laminin α4 (**D**), laminin α5 (**E**), and La4/La5 (**F**). (**G** and **H**) CR and HEV Foxp3^+^ Tregs in pLN (**G**) and mLN (**H**) in no-treatment group (control), Allo only (Allo), and Allo plus rapamycin (Rapa+Allo). (**I**) Heatmap changes in marker expression comparing Allo plus rapamycin to Allo only in pLN and mLN. There were 1–3 LNs/mouse, 3 mice/group, 2–3 sections/LN group, and 7–30 fields/slide. Each dot in the graph represents 1 field. One-way ANOVA. *P < 0.05, **P < 0.01, ***P < 0.001, ****P < 0.0001. Allo, allogeneic splenocytes.

**Figure 7:**
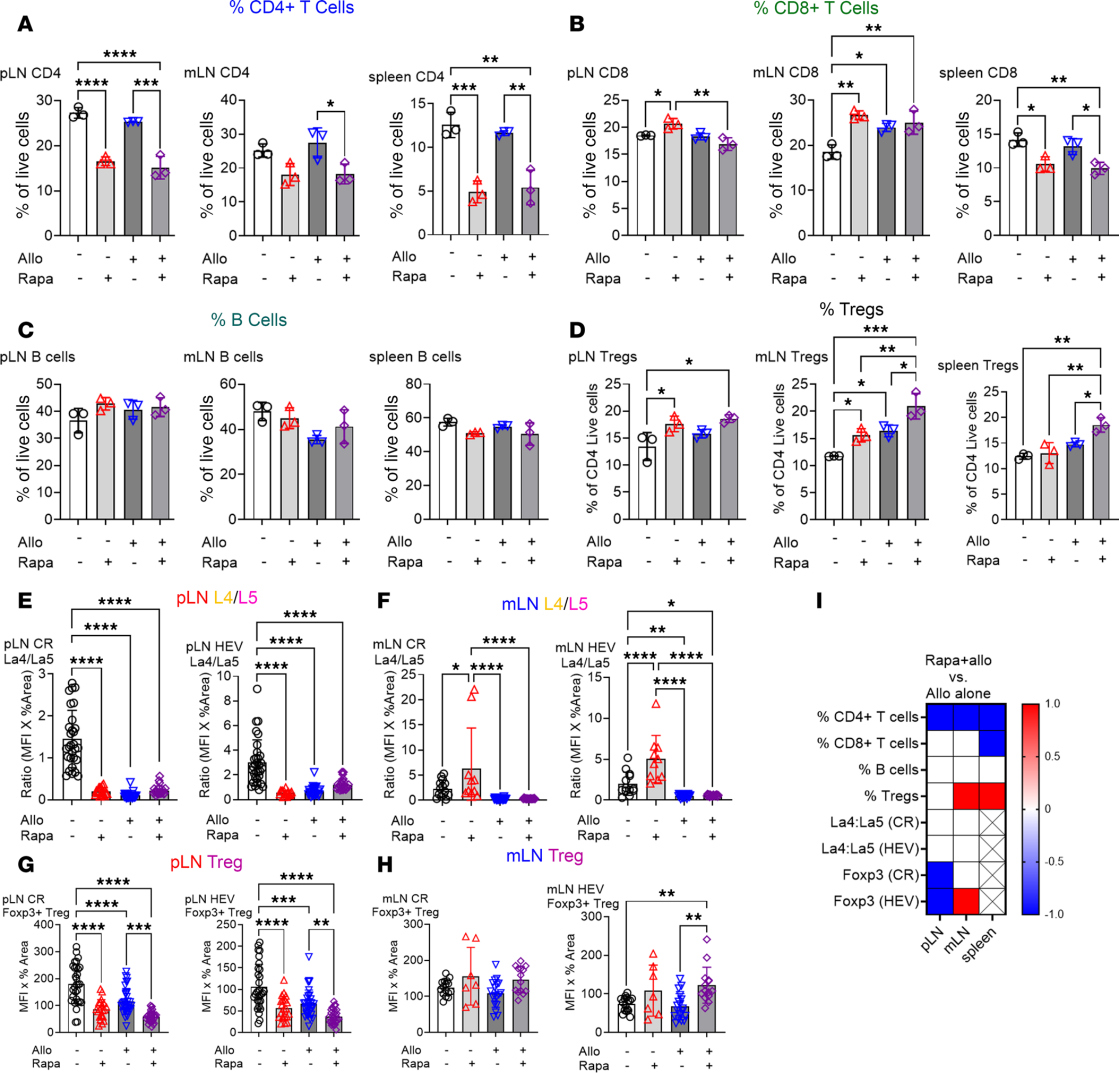
Rapamycin-induced protolerogenic modulation persists in tissue-specific patterns after allogeneic stimulation. Four groups include: untreated B6 mice (control), mice receiving 30 days of rapamycin treatment (Rapa), mice receiving allogeneic stimulation alone with 1 × 10^7^ BALB/c splenocytes i.v. (Allo), or mice receiving 30 days of rapamycin pretreatment followed by allogeneic stimulation (Rapa+Allo). (**A**–**D**) Flow cytometry for percentages of CD4^+^ T cells (**A**), CD8^+^ T cells (**B**), B cells (B220^+^) (**C**), and Foxp3^+^ Tregs (Foxp3^+^CD4^+^) (**D**) in pLN, mLN, and spleen. (**E**–**H**) IHC for La4/La5 in the CR and around HEV in pLN (**E**) and mLN (**F**), and the distribution of Tregs in the CR and HEV in pLN (**G**) and mLN (**H**). There were 1–3 LNs/mouse, 3 mice/group, 2–3 sections/LN group, and 7–30 fields/slide. Each dot in the graph represents 1 field. (**I**) Heatmap of marker expression changes comparing Rapa+Allo to Allo alone in pLN, mLN, and spleen. Red indicates an increase, white indicates no change, blue indicates a decrease, and “X” denotes no data. One-way ANOVA: *P < 0.05, **P < 0.01, ***P < 0.001, ****P < 0.0001.

We next employed a mouse model with allogeneic stimulation (Allo) to characterize the effect of rapamycin on transplant-related alloimmune responses. Mice were injected with fully Allo (1 × 10^7^ cells intravenously) followed by rapamycin treatment for 3 days. Compared with no-treatment control, Allo alone induced a proinflammatory shift by decreasing the La4/La5 in pLNs and mLNs (Figure 6, C and F, and Figure 7, E and F), consistent with our previous findings (51). When Allo was combined with rapamycin (Rapa+Allo), there was an increase in both laminin α4 and α5 compared with Allo alone. However, the increase in laminin α5 exceeded that of laminin α4, resulting in decreased La4/La5 in both pLNs and mLNs (Figure 6, A–F, and I). This pattern aligns with findings from WT and KO mice treated with rapamycin without allostimulation, as both models demonstrated early proinflammatory effect on day 3 (Figure 2 and Figure 4). Compared with no-treatment controls, Allo alone decreased Tregs in the pLN without affecting Tregs in mLN (Figure 6, G–I). After 3 days of Rapa+Allo, compared with Allo alone, there was no change in Treg distribution in pLNs, but Tregs were decreased in the mLNs (Figure 6, G–I), indicating a proinflammatory state. These data demonstrate that rapamycin fosters an early proinflammatory LN environment in the context of alloantigen-induced immune responses through altering La4/La5 and Treg distribution.

**Figure 8:**
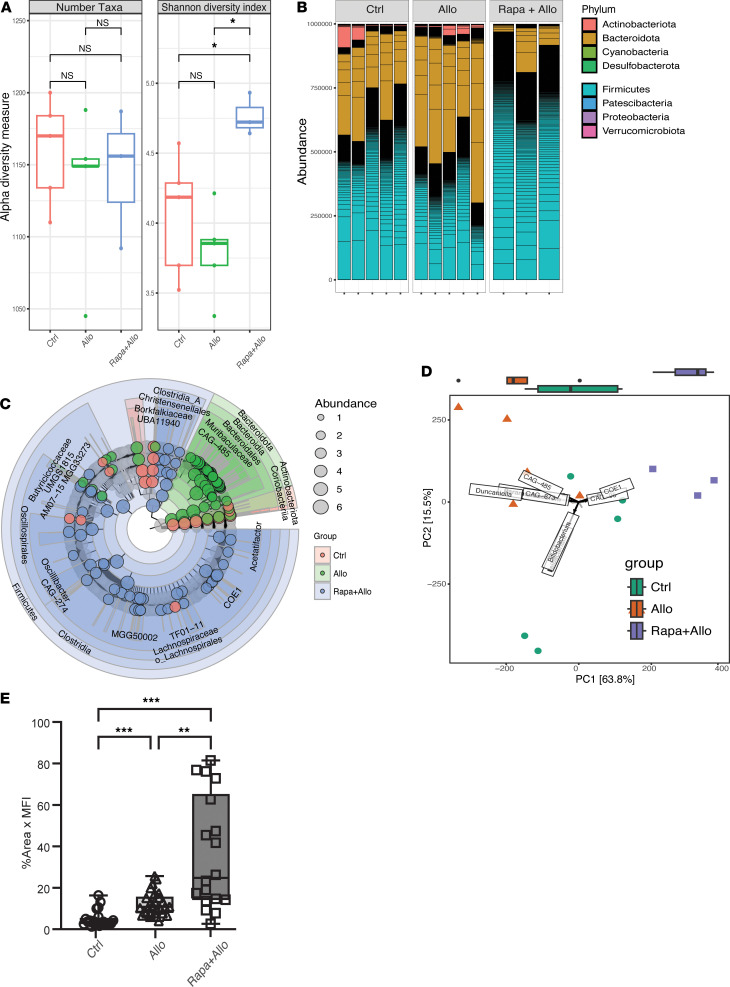
Effects of rapamycin and alloimmunity on gut microbiome and intestinal Foxp3^+^ Treg. (**A**–**C**) Gut microbiome characterization of no-treatment control, allostimulation, and rapamycin combined with allostimulation in diversity index using observed number of taxa and Shannon diversity index (**A**); taxonomic composition on phylum level (**B**); and cladogram of differentially abundant taxonomic groups using LDA effect size (LEfSe) (**C**). Each filled circle represents 1 biomarker. The diameter of a circle is proportional to the phylotype relative abundance scaled at log_10_. (**D**) Principal component analysis (PCA) plot with taxa loadings labeled. Length of each taxa loading vector indicates its contribution to each PCA axis shown. The univariable group distribution appears above the plot. (**E**) IHC of Foxp3 Tregs in intestine assessed after 3 days of rapamycin treatment in allostimulated mice. There were 3 mice/group. O-way ANOVA. *P < 0.05, **P < 0.01, ***P < 0.001. Allo, allogeneic splenocytes.

Rapa+Allo for 3 days led to significant changes in the gut microbiome, characterized by increased microbial diversity and altered community composition and structure. In contrast, Allo alone showed no notable differences compared with the untreated control (Figure 8, A and B, and Supplemental Table 5). While the total number of microbial taxa remained unchanged, the Shannon diversity index increased in the Rapa+Allo group (*P* < 0.05). This suggests that rapamycin, in the context of allostimulation, promotes a more even distribution of microbial species without altering the overall number of distinct taxa. A marked shift in microbiome composition was observed, accompanied by this increase in microbial diversity. The B/F ratio decreased substantially in Rapa+Allo group (0.12 ± 0.10) compared with untreated controls (0.45 ± 0.22) or Allo alone (1.16 ± 0.67), indicating a substantial restructuring of the microbial community (Figure 8B and Supplemental Figure 9A). Allo alone increased the relative abundance of potentially proinflammatory *Muribaculaceae* (i.e., *Duncaniella* and *Paramuribaculum*) within the Bacteroides phylum. However, the combination of Rapa+Allo led to a higher abundance of Firmicutes, including *Lachnospiraceae, Butyricicoccaceae,* and CAG-274 (Figure 8, C and D, and Supplemental Figure 9, B–D). These results demonstrated the rapid, phylogenetic-aware effect of rapamycin under allostimulation where multiple taxa within the same phylogenetic group swiftly shifted in a unified direction. While similar to observations in naive mice under rapamycin treatment, the specific taxonomic groups affected differed. Furthermore, allostimulation increased intestinal Foxp3^+^ Tregs, an effect further enhanced by rapamycin treatment (Figure 8E) mirroring changes seen in naive mice. Collectively, these findings highlight rapamycin’s context-dependent influence on the intestinal microenvironment, affecting both intestinal Treg populations and gut microbiome during allostimulation.

### A tissue-specific persistence of rapamycin-induced tolerogenic effects during allostimulation.

Rapamycin treatment demonstrated its most profound immunomodulatory effects by day 30, establishing a protolerogenic environment characterized by increased Tregs in the pLN and mLN (Supplemental Figure 1, H and I, and Figure 7D), as well as an increased La4/La5 in the mLN (Figure 7, F and I). To evaluate whether this protolerogenic state persists under allostimulation, we conducted experiments comparing 4 groups: untreated control mice, mice receiving 30 days of rapamycin alone (Rapa), mice receiving only allogeneic stimulation with 1 × 10^7^ BALB/c splenocytes i.v. (Allo), and mice receiving 30 days of rapamycin pretreatment followed by allogeneic stimulation (Rapa+Allo). The durability of rapamycin effects showed distinct tissue-specific patterns. In pLNs, the Rapa+Allo group reduced CD4^+^ T cell percentages compared with Allo alone (Figure 7, A and I), while other immune parameters remain largely unchanged (Figure 7, B–E, and I). IHC showed reduced Treg distribution in the Rapa+Allo group (Figure 7, G and I), suggesting that allostimulation partially overcame the rapamycin protolerogenic effect in pLN. This is supported by the observation that Allo alone led to a proinflammatory shift by decreasing the La4/La5 and Treg distribution in pLNs (Figure 6, C and I, and Figure 7, E and G). Together, these findings indicate a partially weakened protolerogenic environment due to allostimulation.

In mLNs, the Rapa+Allo group maintained stronger immunoregulatory features. Rapa+Allo treatment reduced the CD4^+^ T cell percentage compared with Allo only (Figure 7, A and I), with no other changes (Figure 7, B, C, F, and I). Notably, the Rapa+Allo group exhibited an increase in both the percentage and distribution of Tregs (Figure 7, D, H, and I). This preservation of Treg populations and positioning indicates that the 30-day rapamycin pretreatment sustained protolerogenic regulation in mLNs despite allostimulation. The spleen demonstrated the most robust maintenance of a rapamycin-induced protolerogenic state. Rapa+Allo reduced CD4^+^ and CD8^+^ T cell percentages compared with Allo alone (Figure 7, A, B, and I), maintained B cells percentages (Figure 7, C and I), and increased Treg percentages (Figure 7, D and I). Together, these findings reveal that rapamycin established tissue-specific patterns of sustained immune regulation, with the strongest maintenance of protolerogenic features in mLNs and spleen, while pLNs show more susceptibility to allostimulation.

## Discussion

**Figure 9:**
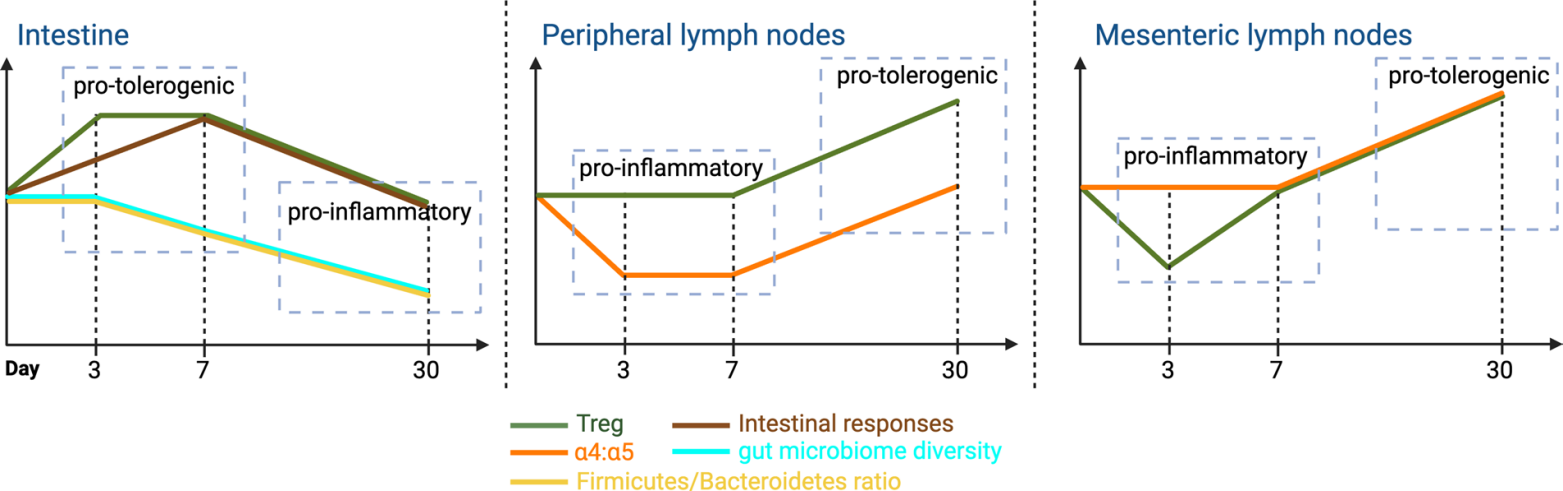
Pattern of changes in intestine, pLN, and mLN. The results of longitudinal changes summarized to demonstrate the temporal changes over time. Illustration created in BioRender (https://BioRender.com/o40n822).

This study was designed to reveal time-dependent and site-specific immunomodulatory effects of rapamycin (Figure 9). The results demonstrated the dynamics of rapamycin influence on immune responses and the gut microbiome, from the intestine to mLNs then pLNs, showing coordinated, multifaceted effect across different anatomical sites and time points. Locally, in the intestine, rapamycin affected gut microbiota, intestinal immune cell responses, and luminal metabolic activities, transitioning from an early protolerogenic to a late proinflammatory state. Regionally, in the mLNs, rapamycin modulated immune responses and LNSC function. The mLNs act as critical intermediaries, reflecting gut-originating changes that affect regional immune responses, demonstrated by dynamic changes in La4/La5 and Treg distribution. Specifically, on day 3, the decrease in overall Tregs, conventional CD4^+^ and CD8^+^ T cells, and Tregs in HEVs and CR suggests a complex immune environment showing both proinflammatory and protolerogenic characteristics. The reduction in Tregs compromises the immune system regulatory capacity, potentially fostering a proinflammatory state due to reduced suppression of effector T cells. Simultaneously, the reduction in effector T cells mitigates the inflammatory response, which channels the environment toward a protolerogenic state, especially in the absence of strong immune activation. This observation suggests that, without additional proinflammatory stimuli, the LN environment on days 3 and 7 hovers between these 2 mixed states, where inflammation is possible but not fully realized. By day 30, the increased overall percentage of Tregs, coupled with an increase in the La4/La5, indicates a more defined protolerogenic shift after continuous rapamycin treatment. These findings highlight the temporal influence of rapamycin, transitioning from an early mixed environment to later predominantly protolerogenic state in both mLNs and pLNs. These findings indicated that rapamycin effects, initiated locally in the gut, rapidly propagated to regional and distant lymphoid tissues and affected overall immune homeostasis. The overlapping effects of rapamycin treatment over time and space illustrate a compartmentalized yet coordinated immune response, emphasizing the need to consider both targeted and broad effects when evaluating immunosuppressive therapies (52).

Rapamycin has well-documented immunosuppressive effects on both innate and adaptive immune responses through the inhibition of mTOR signaling (3, 4). However, mechanisms underlying these effects remain incompletely understood. Our previous research found that laminin α5 inhibits Tregs (51). In this study, we demonstrated that rapamycin effects involve complex interactions between lymphoid architecture and immune cell regulation. Rapamycin facilitated a substantial transition between proinflammatory and protolerogenic states by selectively increasing laminin α5 expression in LNSCs. This finding was validated across naive, laminin α4–KO, laminin α5–KO, and allogeneic splenocyte–stimulated mice. The concurrent changes we observed in overall lymphocyte populations, Treg distribution, and lymphoid architecture suggest that rapamycin orchestrates a coordinated response involving both structural and cellular components of immunity. Rapamycin’s influence on LNSCs likely represents a key mechanism of its action, as LNSCs critically regulate lymphocyte positioning and interaction with APCs via chemokines, cytokines, and stromal fibers (35, 53, 54). By altering LNSC function, rapamycin may reshape the spatial organization and dynamics of immune cell interactions, thereby modulating overall immune responses. This multifaceted effect on structural and cellular aspects of immunity suggests that rapamycin immunomodulatory effects emerge from the integration of multiple mechanisms rather than from isolated changes in a specific immune parameter.

Rapamycin regulation of LN architecture extended to include LECs and BECs. This broad effect on the LNSC network further emphasizes rapamycin’s role in modulating LN function and structure. LECs play a critical role in lymph drainage and immune cell trafficking, while BECs regulate the entry of circulating lymphocytes into the LN. By modulating these cell types, rapamycin could be altering the trafficking patterns and retention of various immune cell subsets within the LN, thereby influencing the initiation and progression of immune responses. Overall, our results highlight its multifaceted mechanisms that act not just on immune cells directly but also on the structural and functional elements of lymphoid organs. This enhanced understanding may inform the analysis of immune mechanisms during the development of new immunosuppressive drugs where precise immune modulation is crucial.

The effects of rapamycin on the gut microbiome were incremental and depended on the duration of exposure. Over time, a temporal shift in intestinal responses and the immune environment was observed, transitioning from suppression to activation following prolonged drug use. There is a growing body of research on rapamycin effects on gut microbiota. Hurez et al. demonstrated that chronic rapamycin treatment altered gut microbiota composition, with notable shifts in Firmicutes and Bacteroidetes abundances that correlated with changes in gut immune cell populations (55). Recent work showed that rapamycin ameliorated experimental colitis through modulation of gut microbiota, particularly by increasing beneficial bacteria like *Lactobacillus reuteri* and reducing potentially harmful species (56). Similarly, chronic mTOR inhibition was shown to particularly affect energy metabolism pathways in the gut microbiome (57). Our study complements these findings by demonstrating how rapamycin progressively reshapes the gut microbial community, with early changes in specific taxa (e.g., *Duncaniella*) followed by broader compositional shifts and metabolic reprogramming. Our findings align with and extend the previous observations by providing a detailed temporal analysis of these alterations, revealing that the most profound changes emerge after prolonged exposure. This time-dependent pattern may explain some variations in previous studies that examined different treatment durations. Our temporal analysis further reveals that these changes coincide with alterations in LN architecture and immune cell trafficking, providing insight into the complex interplay between microbiota and immune regulation under immunosuppression.

One key change was the reprogramming of amino acid metabolism in the gut lumen. This was at least partially due to alterations in the microbiome, specifically a few keystone species that may play a major role in driving these metabolic modifications. Since amino acids are essential for mTORC1 activation, their increased bioavailability in the gut lumen may represent a potential mechanism by which rapamycin influences alloimmune responses. However, the causal relationship between rapamycin treatment, gut microbiome alterations, and immune responses remains unclear. It is yet to be determined whether the observed changes in microbiota composition and function are a direct result of rapamycin effects or an indirect consequence of rapamycin-induced changes in immune and intestinal responses. Future experiments involving immune-deficient mice and fecal microbiota transfer from rapamycin-treated subjects following varied treatment intervals are warranted.

The accumulation of amino acids in the gut lumen during rapamycin-induced immunosuppression is a complex phenomenon attributable to multiple interrelated factors. First, rapamycin inhibition of mTORC1 plays a critical role in regulating protein synthesis (7, 58), as mTORC1 normally promotes translation initiation and protein synthesis through the activation of downstream targets like p70S6 Kinase I (S6K1) and elF4E Binding Protein (4EBP), leading to an accumulation of amino acids that would otherwise be utilized in protein synthesis. Second, mTORC1 inhibition induces autophagy, facilitating the breakdown of intracellular components including proteins by lysosomal degradation, potentially increasing the pool of free amino acids for cell survival (59). Third, rapamycin’s suppression of immune cell proliferation and activity further contributes to amino acid accumulation by decreasing metabolic demand. We observed the reduction in metabolic products like N1-acetylspermidine and the increase in arginine bioavailability, suggesting reduced arginine catabolism due to rapamycin treatment. Fourth, rapamycin disruption of the gut microbiome alters the availability of gut and circulatory amino acids, influencing host nutrient homeostasis and physiology (60). Fifth, rapamycin also directly affects amino acid metabolism in the intestine by modulating synthesis, utilization, and transport processes (61, 62). Further investigation into the metabolic effects of rapamycin could inform targeted nutritional and supportive interventions to optimize rapamycin therapeutic efficacy.

Limitations of our study include that, while our 30-day analysis revealed temporal dynamics in rapamycin effects, this timeframe may not fully capture the long-term changes relevant to transplant recipients on lifelong immunosuppression. The microbiome, intestinal, and immune responses exhibited substantial changes by day 30, but these alterations may continue to evolve over more extended periods, warranting further investigation for clinical relevance. Additionally, our transcriptional analysis identified key DEGs in intestinal responses, but these findings require validation at the translational and functional levels. Future studies should confirm these genes using molecular techniques such as quantitative PCR (qPCR) and protein analysis, particularly in allostimulation models. Such validation would establish reliable molecular signatures of rapamycin effects and identify potential biomarkers for monitoring treatment effect. We also observed discrepancies between IHC and flow cytometry data, likely due to their methodological differences. IHC provides spatial resolution, enabling localized assessments of Tregs within specific areas, whereas flow cytometry offers a quantitative measurement of overall Treg populations. These methods complement each other, highlighting different aspects of Treg dynamics and distribution. Flow cytometry analysis of gut-resident Tregs was hindered by low cell recovery from intestinal tissues, resulting in limited numbers of Foxp3^+^ Tregs (Supplemental Figure 10). However, IHC imaging (Supplemental Figure 7) and quantitative measurements (Figure 5D) revealed that intestinal Foxp3^+^ Treg expression increased with rapamycin treatment on days 3 and 7 but showed no significant differences by day 30. Lastly, our findings on rapamycin’s tissue-specific immune regulation during allostimulation suggest promising applications in transplantation medicine. Optimizing clinical protocols requires further investigation of underlying mechanisms, optimized timing, dosage, and duration of pretreatment, and requires evaluation of its long-term effects on graft survival and immune tolerance. While this study characterizes rapamycin’s effects during primary allostimulation, understanding its effects in sensitized environments represents a crucial next step for transplant medicine. Future research should examine how sensitization affects rapamycin’s immunomodulatory properties, including LN remodeling, immune cell trafficking, and microbiome changes. Of particular interest is how sensitization status influences the gut microbiome, since emerging evidence demonstrates important bidirectional interactions between immune memory and microbiota composition. For example, memory T cells have been shown to regulate intestinal microbiota diversity (11), while specific gut microbes can enhance or suppress memory T cell responses (63, 64). Understanding whether these microbiome changes are driven primarily by the memory immune response, the transplantation procedure itself, or their combined effects will be crucial for optimizing treatment strategies. This knowledge could lead to more effective therapeutic approaches for presensitized transplant recipients, who currently face increased risks of rejection and poorer outcomes.

## Methods

### Sex as a biological variable.

We only used female mice for the current set of experiments where we worked with smaller *n* values, to ensure a high degree of homogeneity within our study groups.

### Mouse experiments.

Female C57BL/6 mice between 8 and 14 weeks of age were purchased from The Jackson Laboratory and were maintained at the University of Maryland School of Medicine Veterinary Resources animal facility. The Pdgfrb-Cre^+/–^ × La4^fl/fl^ (36) and Pdgfrb^–^Cre^+/–^ × La5^fl/fl^ (33) conditional KO mice were previously developed in our laboratory. All mice were cohoused for a minimum of 2 weeks prior to experiments to normalize microbiota. During this period, mice from different treatment groups were housed in the same room and adjacent cages under identical conditions to ensure normalized environmental exposure. At experimental day 0, the mice were randomly separated to different experimental groups and then kept in separate cages to prevent cross exposure. To simulate allostimulation, mice received 1 × 10^7^ fully allogeneic BALB/c splenocytes i.v. on day 0 and were treated with rapamycin daily thereafter. Rapamycin (USP grade, MilliporeSigma) was reconstituted in DMSO (USP grade, MilliporeSigma) at 25 mg/mL. DSMO stock was diluted in sterile PBS to 0.5 mg/mL for i.p. injection at 5 mg/kg/d, following protocols from prior investigations (43, 65). All mice were cohoused and handled together during arrival in the animal facility and for immunosuppressant administration so that the treatment groups were all exposed to each other. On the day of harvest, the mice were euthanized by CO_2_ narcosis, intraluminal stool samples were collected for metabolomic analyses, cardiac puncture was utilized for blood collection, and mLNs, pLNs (axillary, inguinal, and brachial, and cervical), and small intestine were harvested for immunological assays.

### Flow cytometry.

LNs and spleens were disaggregated and passed through 70 μm nylon mesh screens (Thermo Fisher Scientific) to obtain single-cell suspensions. The digestion protocols for FRCs, LECs, and BECs analysis in pLN and mLN were followed as outlined previously (66). The cell suspensions were treated with anti-CD16/32 (clone 93, eBioscience) to block Fc receptors, were stained for 15 minutes at 4°C with antibodies targeting surface molecules (Supplemental Table 1), and were washed 2 times with buffer (PBS with 0.5% w/v bovine serum albumin). Cells were permeabilized using Foxp3/Transcription Factor Staining Buffer Set (eBioscience) according to the manufacturer’s protocol, washed with buffer, and subsequently stained at 4°C with antibodies for intracellular molecules (Supplemental Table 1). Samples were analyzed with an LSR Fortessa Cell Analyzer (BD Biosciences), and data were analyzed using FlowJo software version 10.8.1 (BD Biosciences). Single-color controls (cells stained with single-surface marker antibody) and unstained controls were used for flow channel compensation.

### IHC.

LNs and segments of intestine between the duodenum and jejunum were separately excised and immediately submerged in OCT compound (Sakura Finetek). Sections (6 μm for LNs, 10 μm for intestine) were cut in triplicate using a Microm HM 550 cryostat (Thermo Fisher Scientific) and fixed in cold 1:1 acetone/methanol for 5 minutes and washed in PBS. Primary antibodies (Supplemental Table 1) were diluted between 1:100 and 1:200 in PBST (PBS + 0.03% Triton-X-100 + 0.5% BSA) and incubated for 1 hour in a humidified chamber. Sections were then washed with PBS, and secondary antibodies (Supplemental Table 1) were applied at a 1:400 dilution in PBST for 1 hour. Slides were washed in PBS for 5 minutes, coverslipped, and imaged using an Accu-Scope EXC-500 fluorescent microscope (Nikon), and the images were analyzed with Volocity software (PerkinElmer). For each mouse, 1–2 mLNs, 2–3 pLNs, and 1 piece of intestine were collected. All samples from each treatment group were combined and analyzed as a block, 2–3 sections/block were placed slides, and 7–30 fields/slide were analyzed. The average of mean fluorescence intensity (MFI) for each group was calculated by averaging the MFI values across all slides from all mice, within demarcated regions of LNs and whole intestinal images. Percent area was calculated by dividing the sum area of demarcated regions with marker fluorescence greater than a given threshold by the total analyzed area. Treatment groups were compared using the percentage of area × MFI metric quantification, which incorporte both area and intensity of cell and stromal fiber markers (24).

### Stool specimen collection, DNA extraction, and metagenomic sequencing.

Stool pellets were collected on days 0, 3, and then at weekly intervals and at termination of the experiment for temporal characterization. Intraluminal stools were collected from colon at harvest. They were stored immediately in DNA/RNA Shields (Zymo Research) and archived at –80°C. DNA extraction was described previously (23, 67). In brief, 0.15–0.25 grams of stool samples were extracted using the Quick-DNA Fecal/Soil Microbe kit (Zymo Research). Negative extraction controls were included to ensure that no exogenous DNA contaminated the samples. Metagenomic sequencing libraries were constructed using the Nextera XT Flex Kit (Illumina), according to the manufacturer’s recommendations. Libraries were then pooled together in equimolar proportions and sequenced on a single Illumina NovaSeq 6000 S2 flow cell at Maryland Genomics at the University of Maryland School of Medicine.

### Gut microbiome analyses.

Metagenomic sequence reads mapping to Genome Reference Consortium Mouse Build 39 of strain C57BL/6J (GRCm39) were removed using BMTagger v3.101 (68). Sequence read pairs were removed when 1 or both the read pairs matched the genome reference. The Illumina adapter was trimmed and quality assessment was performed using default parameters in fastp (v.0.21.0). The taxonomic composition of the microbiomes was established using Kraken2 (v.2020.12) and Braken (v. 2.5.0) using the comprehensive mouse microbiota genome catalog (44). Phyloseq R package (v1.38.0) was used to generate the barplot and diversity index. Linear discriminant analysis (LDA) effect size (LEfSe) analysis was used to identify fecal phylotypes that could explain the differences. The α value for the nonparametric factorial Kruskal-Wallis (KW) rank-sum test was set at 0.05, and the threshold for the logarithmic LDA model score for discriminative features was set at 2.0. An all-against-all BLAST search was performed in the multiclass analysis. A phylogram illustrates the taxonomic hierarchy of identified phylotype biomarkers, generated from pairwise group comparisons. The graph was produced using the R package yingtools2 (69). Taxonomic ordination graphs were created with the microViz (v0.12.4). The metagenomic dataset was mapped to the protein database UniRef90 to ensure the comprehensive coverage in functional annotation and was then summarized using HUMAnN3 (Human Microbiome Project Unified Metabolic Analysis Network) (v0.11.2) to determine the presence, absence, and abundance of metabolic pathways in a microbial community. MetaCyc pathway definitions and MinPath were used to identify a parsimonious set of pathways summarized in HUMAnN3 in the microbial community. The total pathway abundance was further stratified by contributing species in HUMAnN3. MaAsLin2 was used to identify the association of the pathways with groups.

### RNA isolation, transcriptome sequencing, and analyses of the intestinal tissues.

The colon tissues of the mice were dissected according to their anatomic features and stored immediately in RNAlater solution (QIAGEN) in RNase-free 1.7 mL tubes (Denville Scientific) at –80°C to stabilize and protect the integrity of RNA. For each sample, total RNA was extracted from approximately 1 cm of ileum. Prior to the extraction, 500 μL of ice-cold RNase-free PBS was added to the sample. To remove the RNAlater, the mixture was centrifuged at 8,000*g* for 10 minutes, and the resulting pellet was resuspended in 500 μL ice-cold RNase-free PBS with 10 μL of β-mercaptoethanol. A tissue suspension was obtained by bead beating procedure using the FastPrep lysing matrix B protocol (MP Biomedicals) to homogenized tissues. RNA was extracted from the resulting suspension using TRIzol Reagent (Invitrogen) following the manufacturer’s recommendations, and this was followed by protein cleanup using Phasemaker tubes (Invitrogen) and precipitation of total nucleic acids using isopropanol. RNA was resuspended in DEPC-treated DNase/RNase-free water. Residual DNA was purged from total RNA extract by treating once with TURBO DNase (Ambion, AM1907) according to the manufacturer’s protocol. DNA removal was confirmed via PCR assay using 16S rRNA primer 27 F (5′-AGAGTTTGATCCTGGCTCAG-3′) and 534 R (5′-CATTACCGCGGCTGCTGG-3′). The quality of extracted RNA was verified using the Agilent 2100 Expert Bioanalyzer using the RNA 1000 Nano kit (Agilent Technologies). Ribosomal RNA depletion and library construction were performed using the RiboZero Plus kit and TruSeq stranded mRNA library preparation kit (Illumina) according to the manufacturer’s recommendations. Libraries were then pooled together in equimolar proportions and sequenced on a single Illumina NovaSeq 6000 S2 flow cell at the Genomic Resource Center (Institute for Genome Sciences, University of Maryland School of Medicine) using the 150 bp paired-end protocol. An average of 87–130 million reads were obtained for each sample. The quality of FASTQ files was evaluated by using FastQC (70). Reads were aligned to the mouse genome (Mus_musculus.GRCm39) using HiSat (version HISAT2-2.0.4), and the number of reads that aligned to the coding regions was determined using HTSeq (71). Significant differential expression was assessed using DEseq2 with an FDR value ≤ 0.05 and fold change (FC) > 2. The overrepresentative analysis was done by importing DEGs against GO ontologies using the enrichGO function of clusterProfile Bioconductor package (72). Only the ontology terms with *q* < 0.05 were used for plotting.

### Metabolome analyses.

Metabolome of intraluminal stool samples collected from ileum was measured using CE/MS to obtain a comprehensive quantitative survey of metabolites (Human Metabolome Technologies). Approximately 10–30 mg of stool was weighed at the time of collection using a company-provided vial and archived at –80°C at the Institute for Genome Sciences at University of Maryland (IGS) until shipped to the Human Metabolome Technologies America (HMT) on dry ice. Quality control procedures included standards, sample blanks, and internal controls that were evenly spaced among the samples analyzed. Compound identification was performed using a capillary electrophoresis mass spectrometry (CE/MS) library of > 1,600 annotated molecules. Log_10_ transformation was applied on data to reduce the influence of measurement noise. Metabolites were annotated using PubChem (48), KEGG (49), and HMDB (50) annotation frameworks that leverage cataloged chemical compounds, known metabolic characterization, and functional hierarchy (i.e., reaction, modules, pathways). The PLS-DA (sparse PLS-DA [sPLS-DA]) algorithm implemented using mixOmics (vers. 6.18.1) was employed to analyze the large dimensional datasets that have more variables (metabolites) than samples (*P* >> *n*) to produce robust and easy-to-interpret models (73). The volcano plot combines results from FC analysis to show significantly increased metabolites after 7-day tacrolimus treatment. A metabolite is shown if FC is > 2 and *P* < 0.05 based on 2-tailed *t* tests. Original metabolite measurements without normalization were used in the FC analysis.

### Statistics.

The experiments were performed in 3 separate trials, with each trial containing at least 3 samples.

Datasets were analyzed using GraphPad Prism 10.2.3. For comparisons of fluorescence markers, immune cell population distribution by flow cytometry, and IHC markers, 2-tailed *t* tests or 1-way ANOVA were used to test for significance defined as *P* < 0.05.

### Study approval.

All procedures involving mice were performed in accordance with the guidelines and regulations set by the Office of Animal Welfare Assurance of the University of Maryland School of Medicine under the approved IACUC protocol nos. 05150001, 0318002, 1220001, and AUP-00000397.

### Data availability.

The data that support the findings of this study are openly available; metagenome sequences were submitted to GenBank under BioProject PRJNA809764 (https://www.ncbi.nlm.nih.gov/bioproject/PRJNA809764) with the SRA study ID SRP361281. The R codes, including each step and parameters, were deposited in GitHub at https://github.com/igsbma/rapamycin.git RNA-Seq data were deposited in the NCBI Gene Expression Omnibus database (accession no. GSE288851). The preprint associated with this study is available at https://www.biorxiv.org/content/10.1101/2024.10.01.616121v1

Qualitative heatmaps were generated (GraphPad prism) to express changes in marker expression level relative to control using 1 to represent “increased,” 0 to represent “unchanged,” and –1 to represent “decreased.” All raw data can be found in the Supporting Data Values file.

## Author contributions

LW, BM, and JSB designed the experiments. LW, AK, SJG, RL, DK, LL, VS, WP, and MWS conducted and analyzed the in vitro and in vivo experiments. HWL performed nucleic acid extraction and sequencing library preparation. BM and YS performed the bioinformatics analyses. BM, LW, MTF, SJG, VRM, and JSB wrote the manuscript. BM and JSB are cocorresponding authors for this manuscript.

## Supplementary Material

Supplemental data

Supplemental table 2

Supplemental table 3

Supplemental table 4

Supplemental table 5

Supporting data values

## Version 1 04/22/2025

Electronic publication
