# Supplementary material for: Rapamycin immunomodulation utilizes time-dependent alterations of lymph node architecture, leukocyte trafficking, and gut microbiome
Source: JCI Insight. 2025 Apr 22;10(8):e186505. doi: 10.1172/jci.insight.186505 (PMC12016939; doi:10.1172/jci.insight.186505)
Supplement: Supplemental data [file jciinsight-10-186505-s122.pdf]

Supplemental Figures

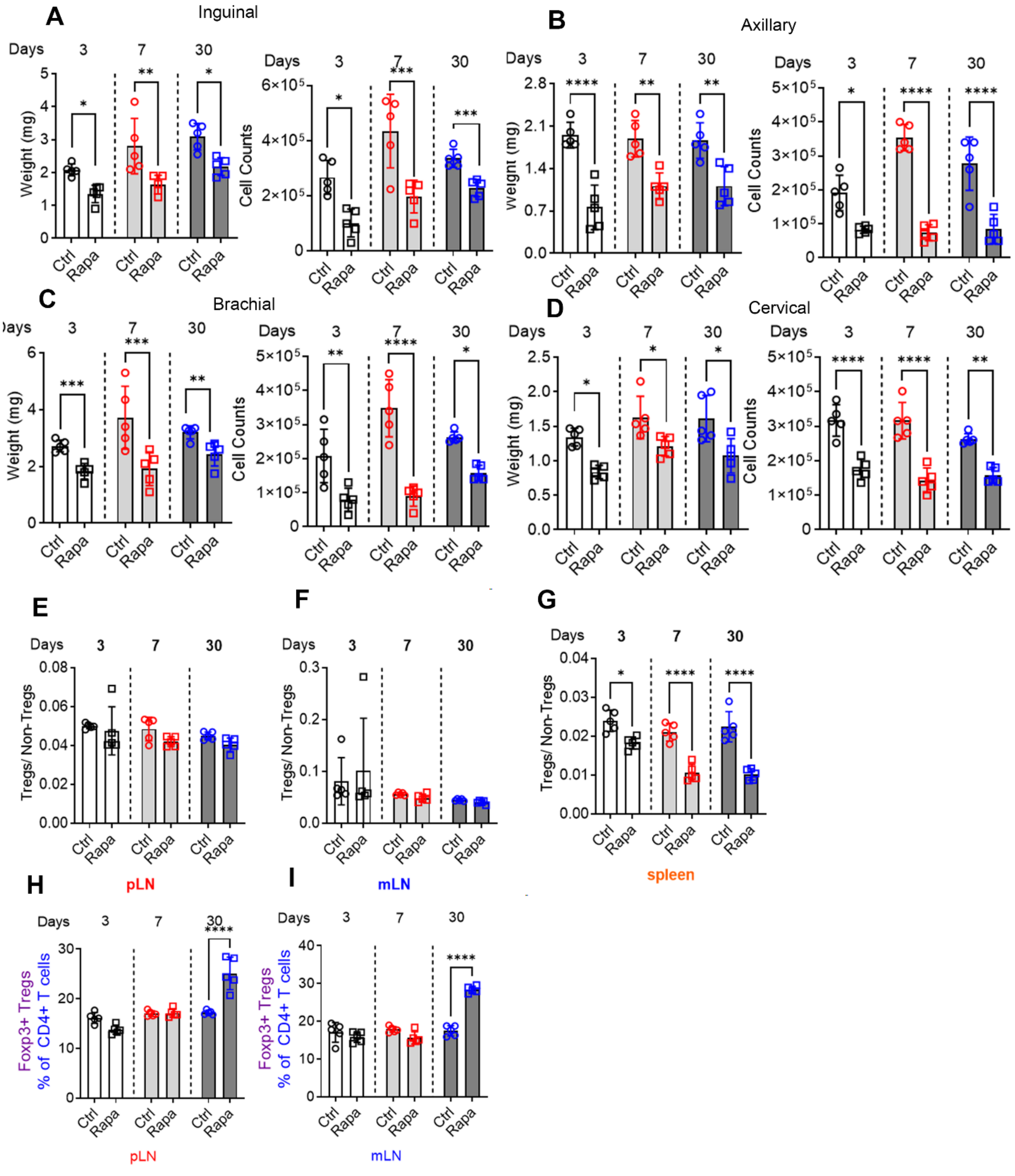

Supplemental Figure 1. Rapamycin reduces cell counts in pLN. Weight and cell number for **A**) inguinal, **B)** axillary, **C)** brachial, and **D)** cervical LNs. Ratios of Tregs to non-Tregs in the **E)** pLN, **F)** mLN, and **G)**

spleen. Flow cytometry analysis for percentage of Foxp3+ Tregs in CD4+ T cells in **H)** pLN and **I)** mLN at days 3, 7, and 30 post-rapamycin treatment. 5 mice/group. One-way ANOVA. *p<0.05, **p<0.01, ** p<0.001, ****p<0.0001.

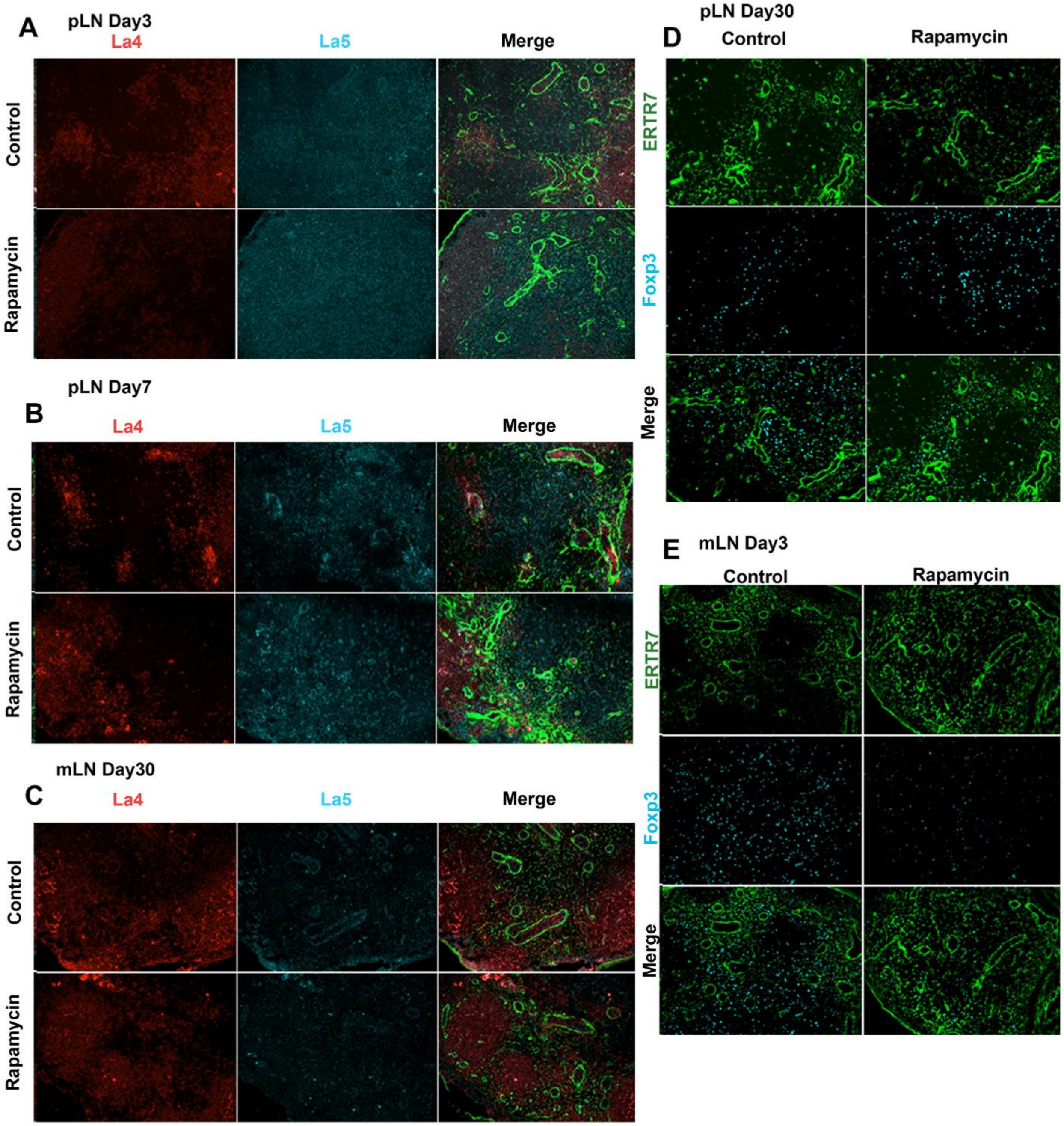

Supplemental Figure 2. Effects of rapamycin on LN cell content, cell distribution, and structure. IHC images of Laminin α4, Laminin α5 for **A**) pLN on day 3, **B)** pLN on day 7, and **C)** mLN on day 30. ERTR7, Foxp3 for **D**) pLN on day 30 and **E**) mLN on day 3.

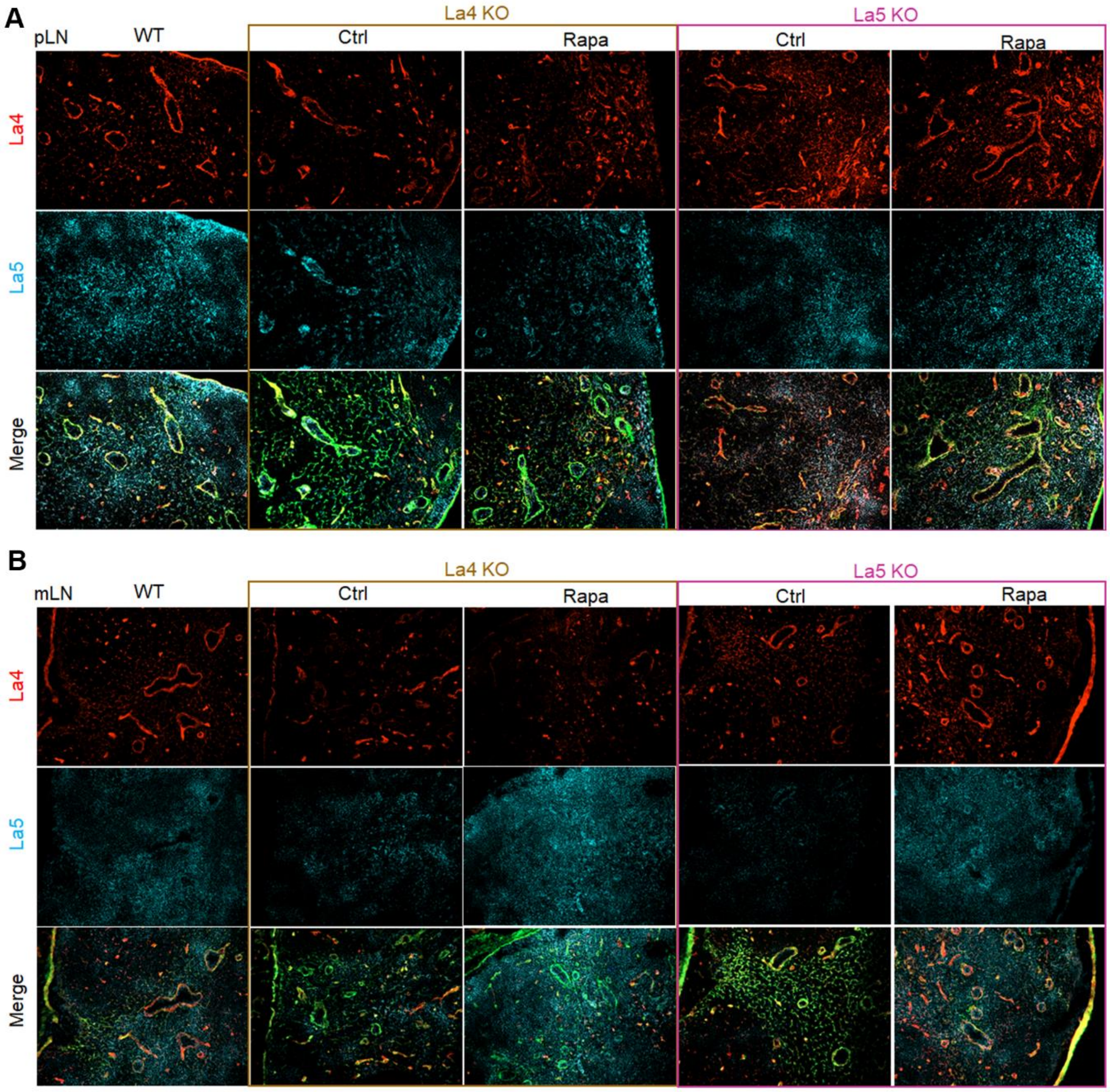

Supplemental Figure 3. Rapamycin regulates laminin α5 in FRC-Lama4-KO and FRC-Lama5-KO mice. IHC images of Laminin α4, Laminin α5 for **A)** pLN and **B)** mLN on WT, FRC-Lama4-KO and FRC-Lama5-KO mice.

A

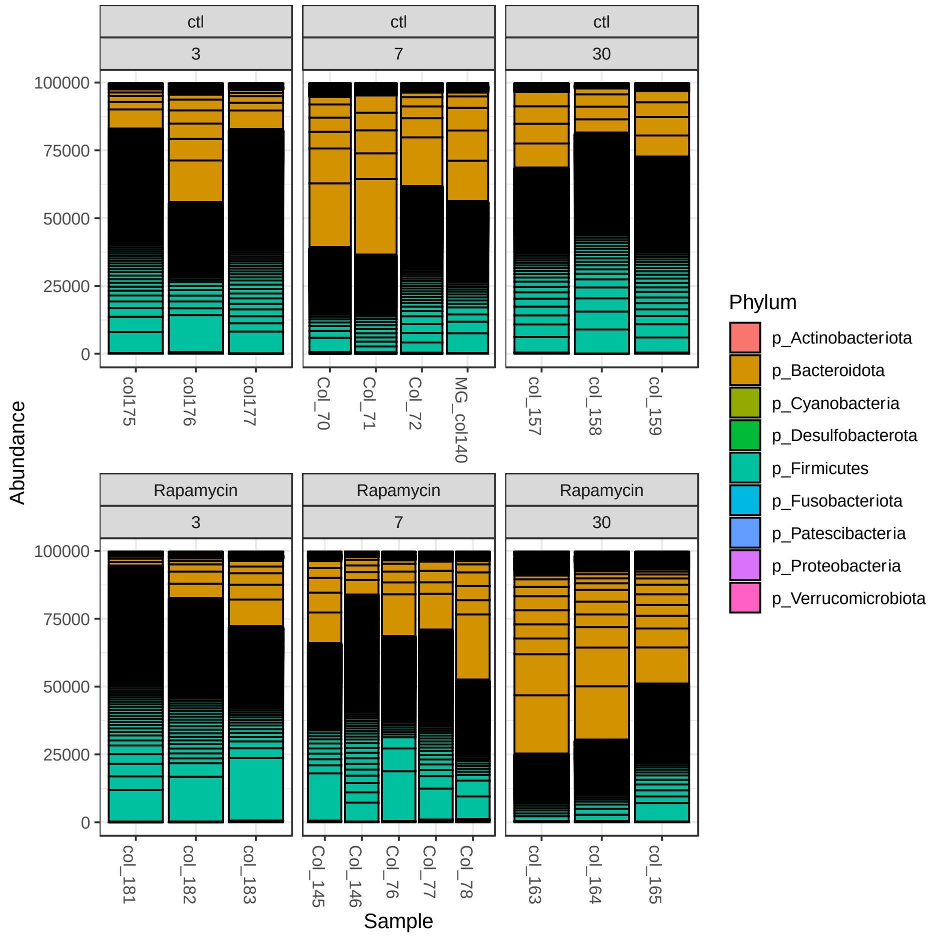

ctl
3
7
30
Rapamycin
Abundance
Sample
100000
75000
50000
25000
0
col175
col176
col177
Col_70
Col_71
Col_72
MG_col140
col_157
col_158
col_159
col_181
col_182
col_183
Col_145
Col_146
Col_76
Col_77
Col_78
col_163
col_164
col_165
Phylum
p_Actinobacteriota
p_Bacteroidota
p_Cyanobacteria
p_Desulfobacterota
p_Firmicutes
p_Fusobacteriota
p_Patescibacteria
p_Proteobacteria
p_Verrucomicrobiota

B

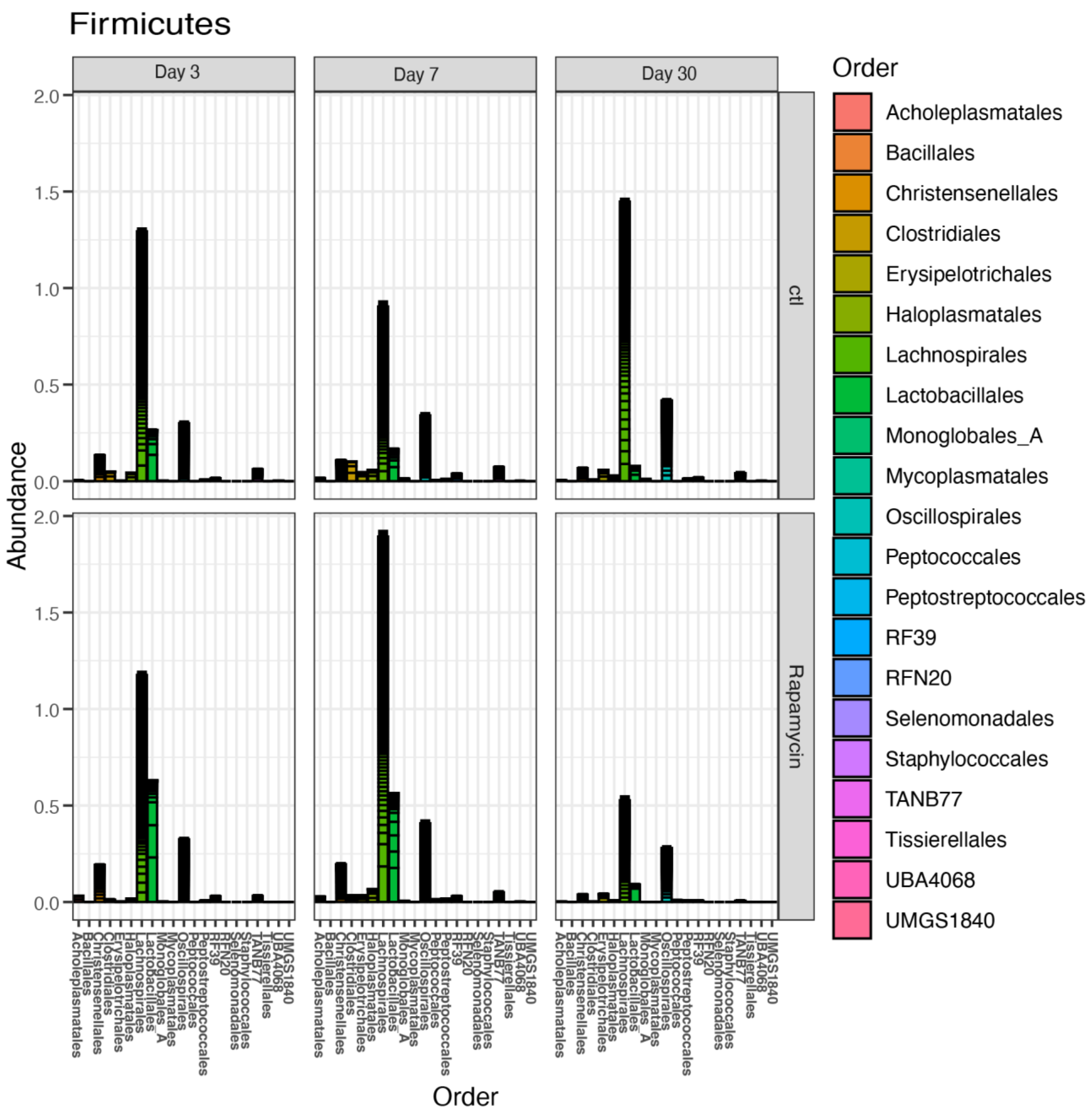

Firmicutes
Day 3
Day 7
Day 30
ctl
Rapamycin
Abundance
2.0
1.5
1.0
0.5
0.0
Order
Acholeplasmatales
Bacillales
Christensenellales
Clostridiales
Erysipelotrichales
Haloplasmatales
Lachnospirales
Lactobacillales
Monoglobales_A
Mycoplasmatales
Oscillospirales
Peptococcales
Peptostreptococcales
RF39
RFN20
Selenomonadales
Staphylococcales
TANB77
Tissierellales
UBA4068
UMGS1840

C

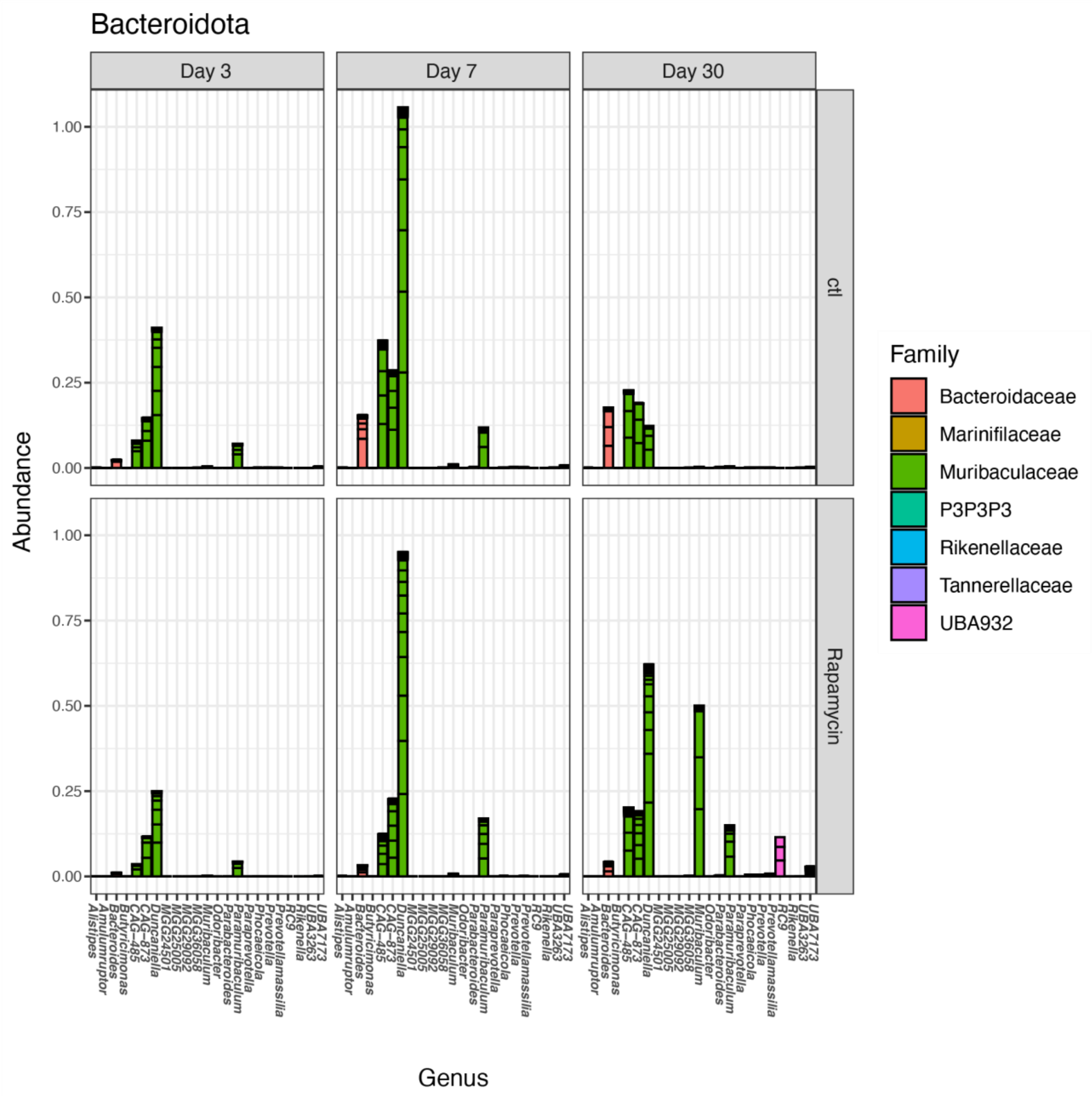
Bacteroidota
Day 3
Day 7
Day 30
ctl
Rapamycin
Abundance
0.00
0.25
0.50
0.75
1.00
Genus
Alistipes
Amulumruptor
Bacteroides
Butyricimonas
CAG−485
CAG−873
Duncaniella
MGG24501
MGG25005
MGG29092
MGG36058
Muribaculum
Odoribacter
Parabacteroides
Paramuribaculum
Paraprevotella
Phocaeicola
Prevotella
Prevotellamassilia
RC9
Rikenella
UBA3263
UBA7173
Family
Bacteroidaceae
Marinifilaceae
Muribaculaceae
P3P3P3
Rikenellaceae
Tannerellaceae
UBA932

D

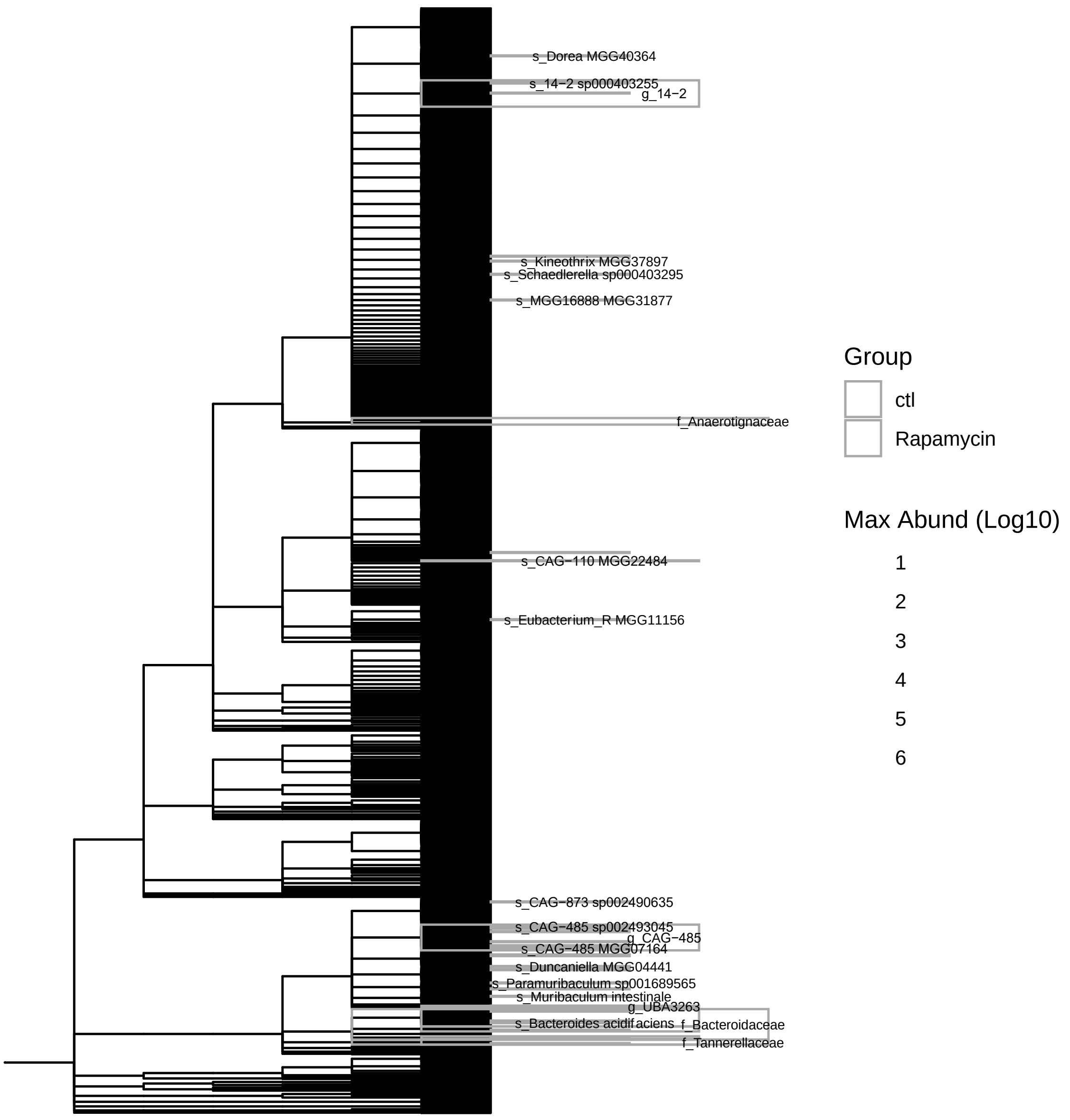
s_Dorea MGG40364
s_14−2 sp000403255
g_14−2
s_Kineothrix MGG37897
s_Schaedlerella sp000403295
s_MGG16888 MGG31877
f_Anaerotignaceae
s_CAG−110 MGG22484
s_Eubacterium_R MGG11156
s_CAG−873 sp002490635
s_CAG−485 sp002493045
g_CAG−485
s_CAG−485 MGG07164
s_Duncaniella MGG04441
s_Paramuribaculum sp001689565
s_Muribaculum intestinale
g_UBA3263
s_Bacteroides acidifaciens
f_Bacteroidaceae
f_Tannerellaceae
Group
ctl
Rapamycin
Max Abund (Log10)
1
2
3
4
5
6

E

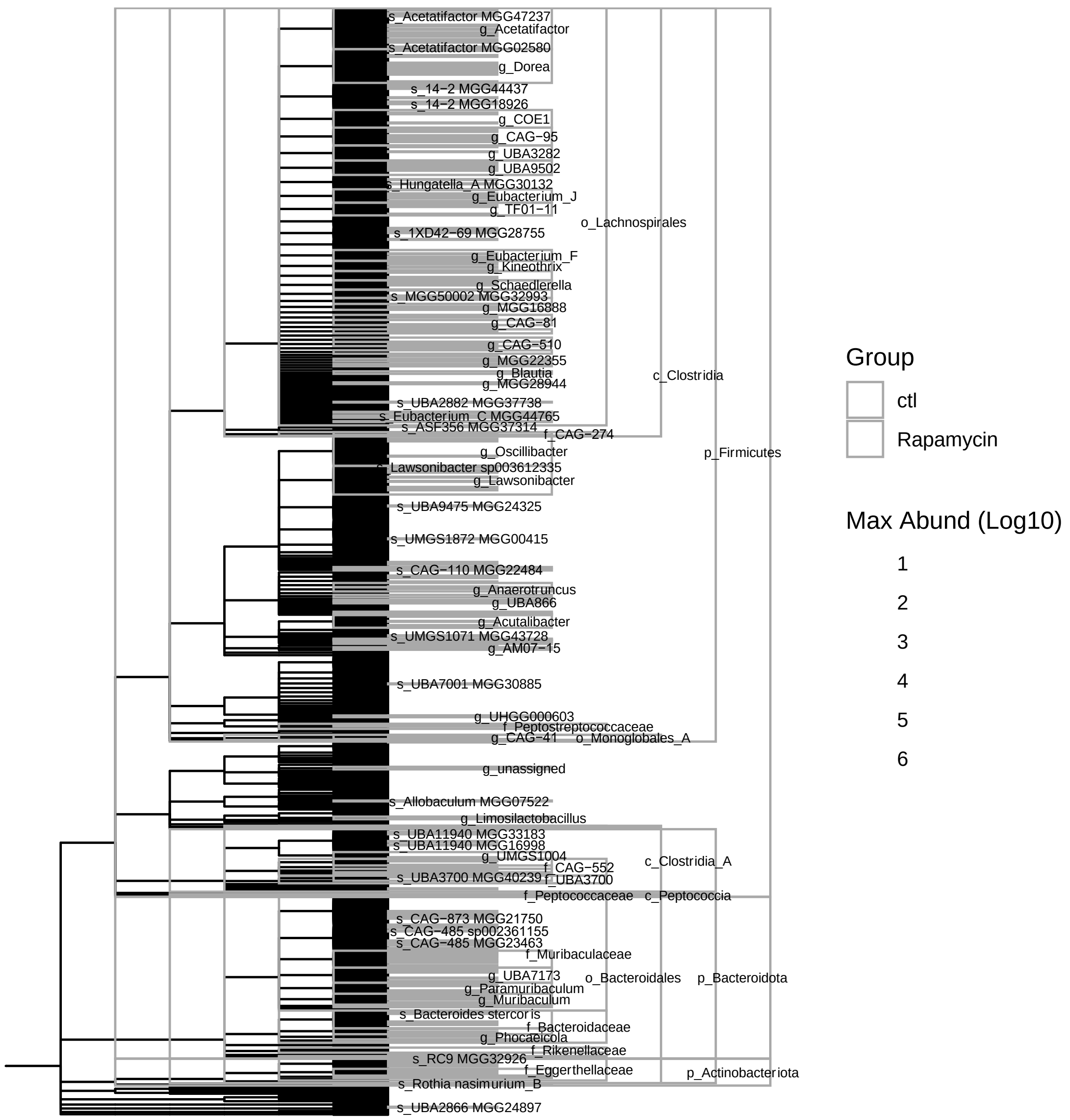

Supplemental Figure 4. Rapamycin increases Firmicute of gut microbiome. A) Taxonomic composition of gut microbiome at phylum level after rapamycin treatment for 3, 7 and 30 days compared to no treatment control. Cumulative relative abundance of **B**) Firmicutes, **C**) Bacteroidota to compare relative abundance at genus level. Logarithmic linear discriminant analysis (LDA) effect size (LEfSe) (Segata et al., 2011) of the identified phylotype biomarkers of **D)** 7-day treatment; and **E**) 30-day treatment.

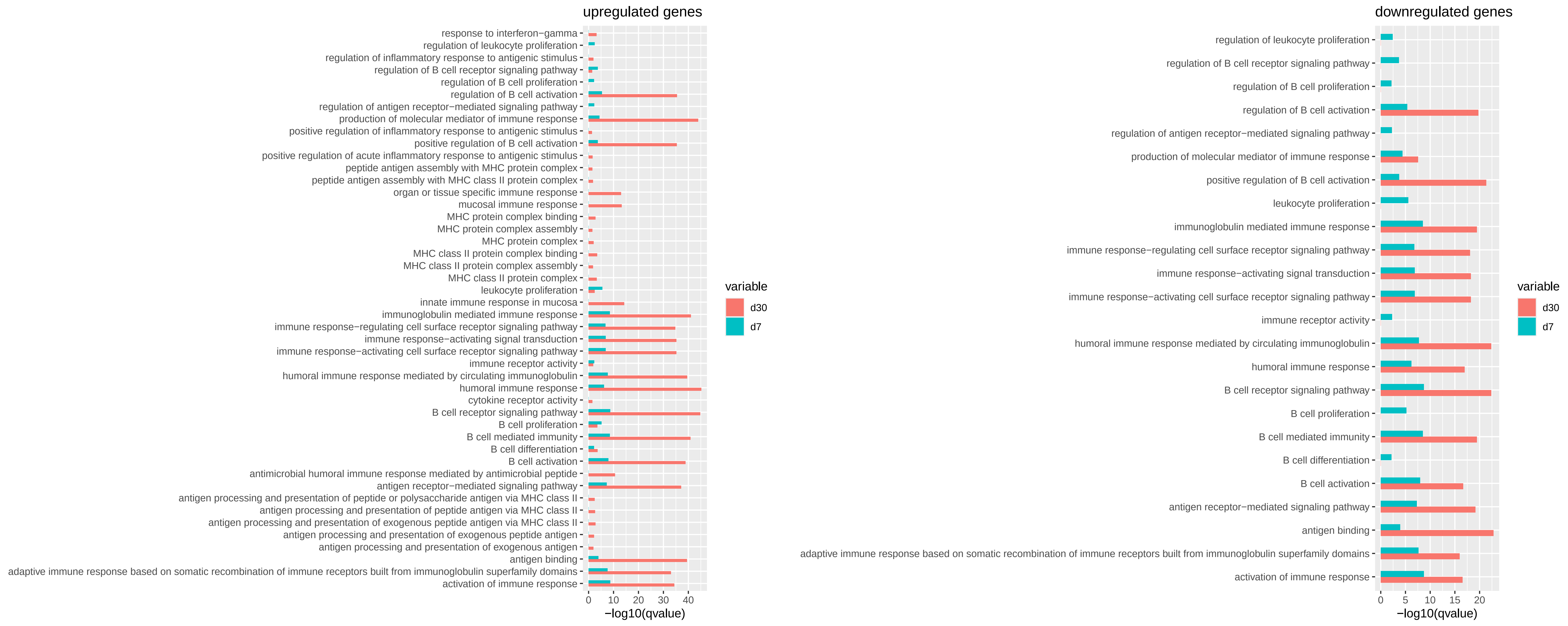

upregulated genes
response to interferon−gamma
regulation of leukocyte proliferation
regulation of inflammatory response to antigenic stimulus
regulation of B cell receptor signaling pathway
regulation of B cell proliferation
regulation of B cell activation
regulation of antigen receptor−mediated signaling pathway
production of molecular mediator of immune response
positive regulation of inflammatory response to antigenic stimulus
positive regulation of B cell activation
positive regulation of acute inflammatory response to antigenic stimulus
peptide antigen assembly with MHC protein complex
peptide antigen assembly with MHC class II protein complex
organ or tissue specific immune response
mucosal immune response
MHC protein complex binding
MHC protein complex assembly
MHC protein complex
MHC class II protein complex binding
MHC class II protein complex assembly
MHC class II protein complex
leukocyte proliferation
innate immune response in mucosa
immunoglobulin mediated immune response
immune response−regulating cell surface receptor signaling pathway
immune response−activating signal transduction
immune response−activating cell surface receptor signaling pathway
immune receptor activity
humoral immune response mediated by circulating immunoglobulin
humoral immune response
cytokine receptor activity
B cell receptor signaling pathway
B cell proliferation
B cell mediated immunity
B cell differentiation
B cell activation
antimicrobial humoral immune response mediated by antimicrobial peptide
antigen receptor−mediated signaling pathway
antigen processing and presentation of peptide or polysaccharide antigen via MHC class II
antigen processing and presentation of peptide antigen via MHC class II
antigen processing and presentation of exogenous peptide antigen via MHC class II
antigen processing and presentation of exogenous peptide antigen
antigen processing and presentation of exogenous antigen
antigen binding
adaptive immune response based on somatic recombination of immune receptors built from immunoglobulin superfamily domains
activation of immune response
0 10 20 30 40
−log10(qvalue)
variable
d30
d7
downregulated genes
regulation of leukocyte proliferation
regulation of B cell receptor signaling pathway
regulation of B cell proliferation
regulation of B cell activation
regulation of antigen receptor−mediated signaling pathway
production of molecular mediator of immune response
positive regulation of B cell activation
leukocyte proliferation
immunoglobulin mediated immune response
immune response−regulating cell surface receptor signaling pathway
immune response−activating signal transduction
immune response−activating cell surface receptor signaling pathway
immune receptor activity
humoral immune response mediated by circulating immunoglobulin
humoral immune response
B cell receptor signaling pathway
B cell proliferation
B cell mediated immunity
B cell differentiation
B cell activation
antigen receptor−mediated signaling pathway
antigen binding
adaptive immune response based on somatic recombination of immune receptors built from immunoglobulin superfamily domains
activation of immune response
0 5 10 15 20
−log10(qvalue)
variable
d30
d7

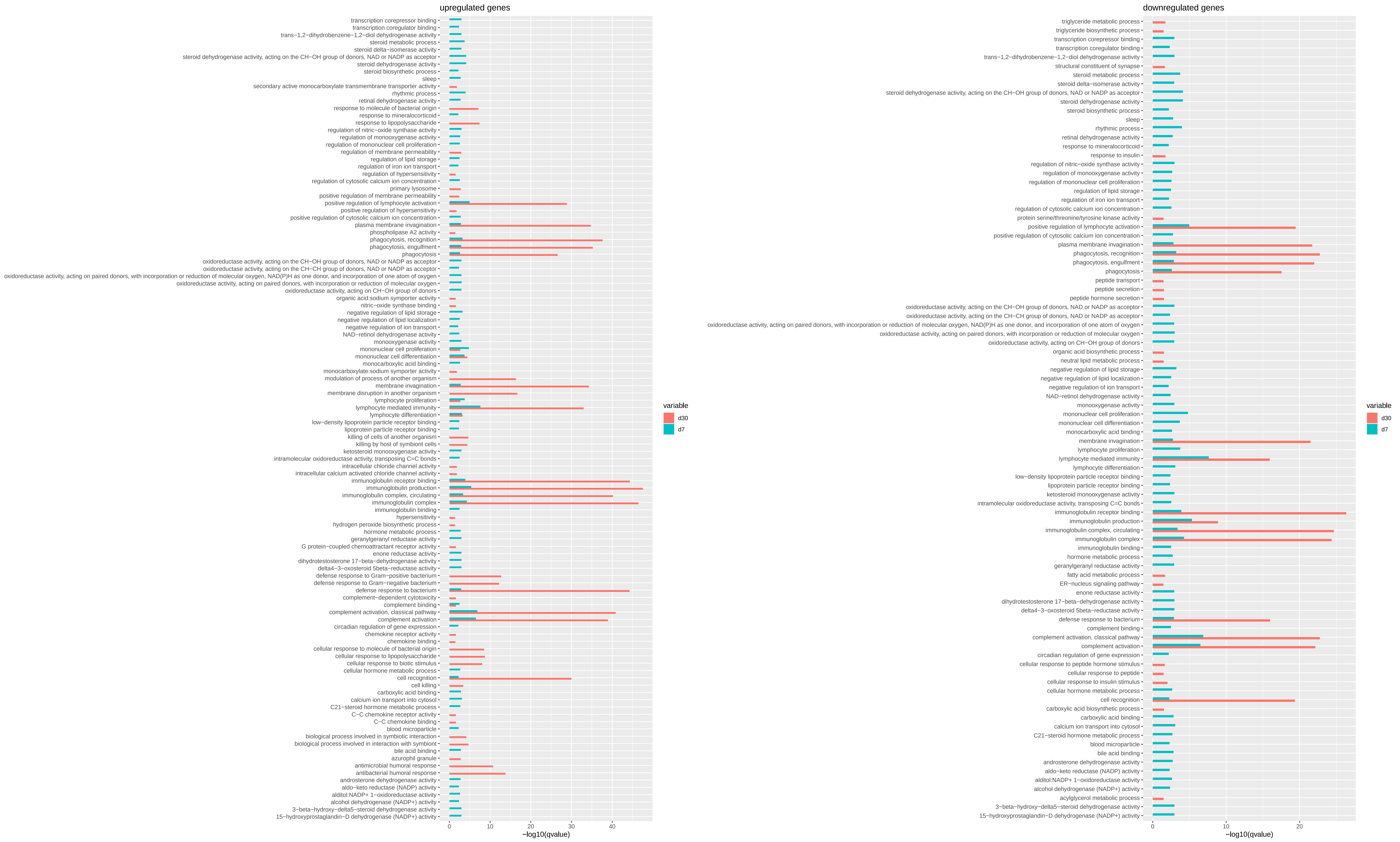
upregulated genes
transcription corepressor binding
transcription coregulator binding
trans−1,2−dihydrobenzene−1,2−diol dehydrogenase activity
steroid metabolic process
steroid delta−isomerase activity
steroid dehydrogenase activity, acting on the CH−OH group of donors, NAD or NADP as acceptor
steroid dehydrogenase activity
steroid biosynthetic process
sleep
secondary active monocarboxylate transmembrane transporter activity
rhythmic process
retinal dehydrogenase activity
response to molecule of bacterial origin
response to mineralocorticoid
response to lipopolysaccharide
regulation of nitric−oxide synthase activity
regulation of monooxygenase activity
regulation of mononuclear cell proliferation
regulation of membrane permeability
regulation of lipid storage
regulation of iron ion transport
regulation of hypersensitivity
regulation of cytosolic calcium ion concentration
primary lysosome
positive regulation of membrane permeability
positive regulation of lymphocyte activation
positive regulation of hypersensitivity
positive regulation of cytosolic calcium ion concentration
plasma membrane invagination
phospholipase A2 activity
phagocytosis, recognition
phagocytosis, engulfment
phagocytosis
oxidoreductase activity, acting on the CH−OH group of donors, NAD or NADP as acceptor
oxidoreductase activity, acting on the CH−CH group of donors, NAD or NADP as acceptor
oxidoreductase activity, acting on paired donors, with incorporation or reduction of molecular oxygen, NAD(P)H as one donor, and incorporation of one atom of oxygen
oxidoreductase activity, acting on paired donors, with incorporation or reduction of molecular oxygen
oxidoreductase activity, acting on CH−OH group of donors
organic acid:sodium symporter activity
nitric−oxide synthase binding
negative regulation of lipid storage
negative regulation of lipid localization
negative regulation of ion transport
NAD−retinol dehydrogenase activity
monooxygenase activity
mononuclear cell proliferation
mononuclear cell differentiation
monocarboxylic acid binding
monocarboxylate:sodium symporter activity
modulation of process of another organism
membrane invagination
membrane disruption in another organism
lymphocyte proliferation
lymphocyte mediated immunity
lymphocyte differentiation
low−density lipoprotein particle receptor binding
lipoprotein particle receptor binding
killing of cells of another organism
killing by host of symbiont cells
ketosteroid monooxygenase activity
intramolecular oxidoreductase activity, transposing C=C bonds
intracellular chloride channel activity
intracellular calcium activated chloride channel activity
immunoglobulin receptor binding
immunoglobulin production
immunoglobulin complex, circulating
immunoglobulin complex
immunoglobulin binding
hypersensitivity
hydrogen peroxide biosynthetic process
hormone metabolic process
geranylgeranyl reductase activity
G protein−coupled chemoattractant receptor activity
enone reductase activity
dihydrotestosterone 17−beta−dehydrogenase activity
delta4−3−oxosteroid 5beta−reductase activity
defense response to Gram−positive bacterium
defense response to Gram−negative bacterium
defense response to bacterium
complement−dependent cytotoxicity
complement binding
complement activation, classical pathway
complement activation
circadian regulation of gene expression
chemokine receptor activity
chemokine binding
cellular response to molecule of bacterial origin
cellular response to lipopolysaccharide
cellular response to biotic stimulus
cellular hormone metabolic process
cell recognition
cell killing
carboxylic acid binding
calcium ion transport into cytosol
C21−steroid hormone metabolic process
C−C chemokine receptor activity
C−C chemokine binding
blood microparticle
biological process involved in symbiotic interaction
biological process involved in interaction with symbiont
bile acid binding
azurophil granule
antimicrobial humoral response
antibacterial humoral response
androsterone dehydrogenase activity
aldo−keto reductase (NADP) activity
alditol:NADP+ 1−oxidoreductase activity
alcohol dehydrogenase (NADP+) activity
3−beta−hydroxy−delta5−steroid dehydrogenase activity
15−hydroxyprostaglandin−D dehydrogenase (NADP+) activity
0
10
20
30
40
−log10(qvalue)
variable
d30
d7
downregulated genes
triglyceride metabolic process
triglyceride biosynthetic process
transcription corepressor binding
transcription coregulator binding
trans−1,2−dihydrobenzene−1,2−diol dehydrogenase activity
structural constituent of synapse
steroid metabolic process
steroid delta−isomerase activity
steroid dehydrogenase activity, acting on the CH−OH group of donors, NAD or NADP as acceptor
steroid dehydrogenase activity
steroid biosynthetic process
sleep
rhythmic process
retinal dehydrogenase activity
response to mineralocorticoid
response to insulin
regulation of nitric−oxide synthase activity
regulation of monooxygenase activity
regulation of mononuclear cell proliferation
regulation of lipid storage
regulation of iron ion transport
regulation of cytosolic calcium ion concentration
protein serine/threonine/tyrosine kinase activity
positive regulation of lymphocyte activation
positive regulation of cytosolic calcium ion concentration
plasma membrane invagination
phagocytosis, recognition
phagocytosis, engulfment
phagocytosis
peptide transport
peptide secretion
peptide hormone secretion
oxidoreductase activity, acting on the CH−OH group of donors, NAD or NADP as acceptor
oxidoreductase activity, acting on the CH−CH group of donors, NAD or NADP as acceptor
oxidoreductase activity, acting on paired donors, with incorporation or reduction of molecular oxygen, NAD(P)H as one donor, and incorporation of one atom of oxygen
oxidoreductase activity, acting on paired donors, with incorporation or reduction of molecular oxygen
oxidoreductase activity, acting on CH−OH group of donors
organic acid biosynthetic process
neutral lipid metabolic process
negative regulation of lipid storage
negative regulation of lipid localization
negative regulation of ion transport
NAD−retinol dehydrogenase activity
monooxygenase activity
mononuclear cell proliferation
mononuclear cell differentiation
monocarboxylic acid binding
membrane invagination
lymphocyte proliferation
lymphocyte mediated immunity
lymphocyte differentiation
low−density lipoprotein particle receptor binding
lipoprotein particle receptor binding
ketosteroid monooxygenase activity
intramolecular oxidoreductase activity, transposing C=C bonds
immunoglobulin receptor binding
immunoglobulin production
immunoglobulin complex, circulating
immunoglobulin complex
immunoglobulin binding
hormone metabolic process
geranylgeranyl reductase activity
fatty acid metabolic process
ER−nucleus signaling pathway
enone reductase activity
dihydrotestosterone 17−beta−dehydrogenase activity
delta4−3−oxosteroid 5beta−reductase activity
defense response to bacterium
complement binding
complement activation, classical pathway
complement activation
circadian regulation of gene expression
cellular response to peptide hormone stimulus
cellular response to peptide
cellular response to insulin stimulus
cellular hormone metabolic process
cell recognition
carboxylic acid biosynthetic process
carboxylic acid binding
calcium ion transport into cytosol
C21−steroid hormone metabolic process
blood microparticle
bile acid binding
androsterone dehydrogenase activity
aldo−keto reductase (NADP) activity
alditol:NADP+ 1−oxidoreductase activity
alcohol dehydrogenase (NADP+) activity
acylglycerol metabolic process
3−beta−hydroxy−delta5−steroid dehydrogenase activity
15−hydroxyprostaglandin−D dehydrogenase (NADP+) activity
0
10
20
−log10(qvalue)
variable
d30
d7

Supplemental Figure 5. Rapamycin effect on the functional pathways of gut microbiome and contributing species. Significantly enriched functional pathways in no treatment control group and rapamycin group, and responsible taxonomic groups in gut microbiome. Functional pathway composition and abundance generated using UniRef90 (1) and HUMAnN3 (2) following MetaCyc pathway definitions (3) and MinPath (4), and resulting pathways were stratified by contributing species. Figures are available to download at https://doi.org/10.6084/m9.figshare.26343112.

A

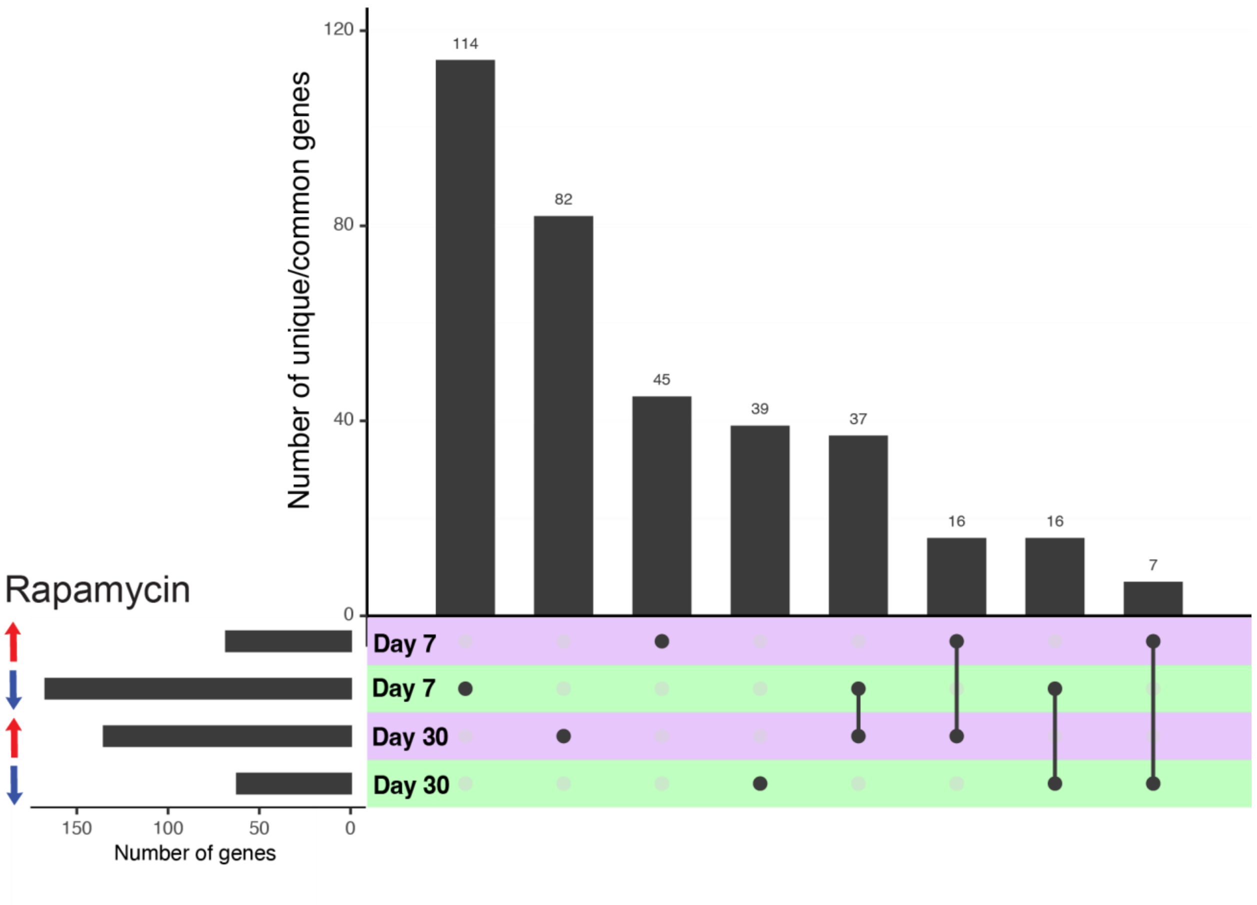

Supplemental Figure 6. Analysis of differentially expressed genes (DEGs) after rapamycin treatment for 7 and 30 days. A) UpSet plots showing intersections of DEGs for each treatment compared to control. Top enriched **B**) immune-related and **C**) non-immune functional pathways of differential expressed genes of small intestinal tissues in rapamycin treated group after 7 and 30 days compared to no treatment control. Significant differential expression assessed using DEseq2 (5), the over-representative analysis performed by importing Differentially Expressed Genes (DEGs) against GO ontologies using the enrichGO function (6). Figures of B and C are available to download at https://doi.org/10.6084/m9.figshare.27922197.

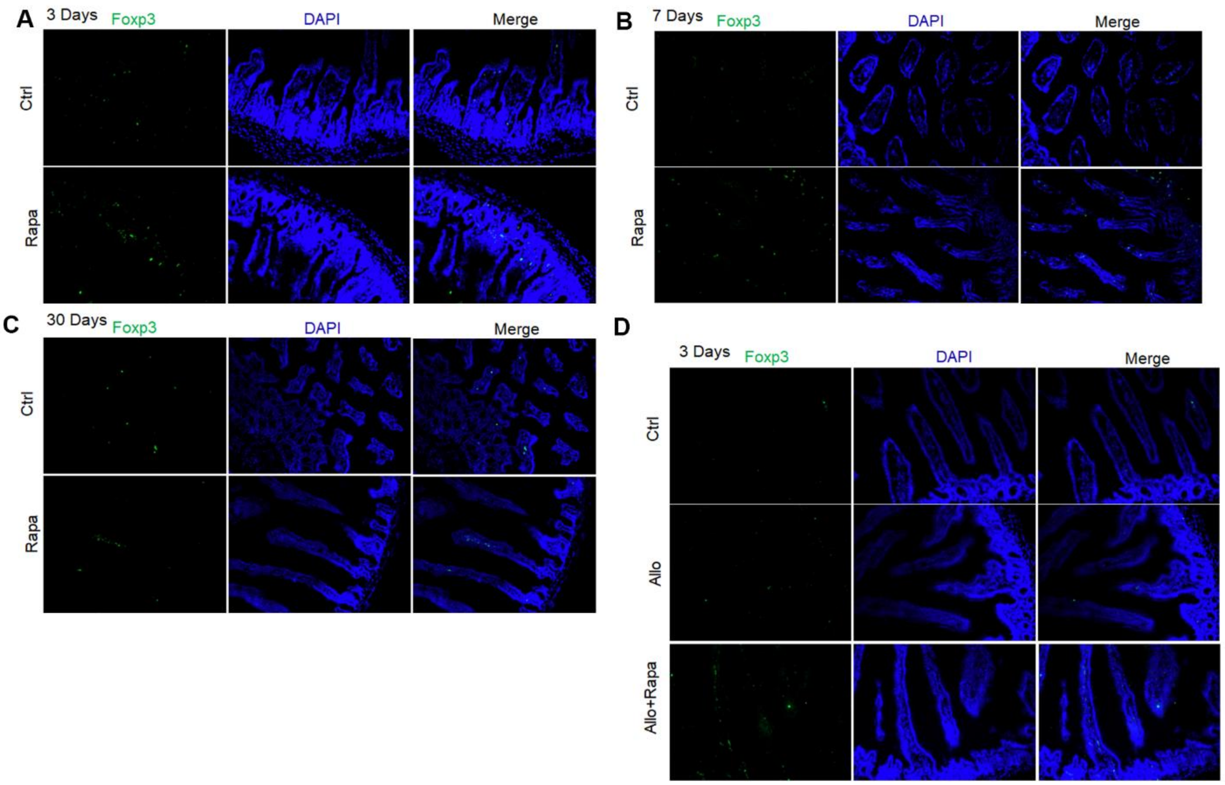

Supplemental Figure 7. Rapamycin impacts intestinal Foxp3 Tregs. IHC images showingTregs in the intestine at **A)** day 3, **B)** day 7 and **C)** day 30 for the no-treatment and rapamycin-treated groups, and **D)** day 3 for the no-treatment, allo, and rapamycin + allo groups.

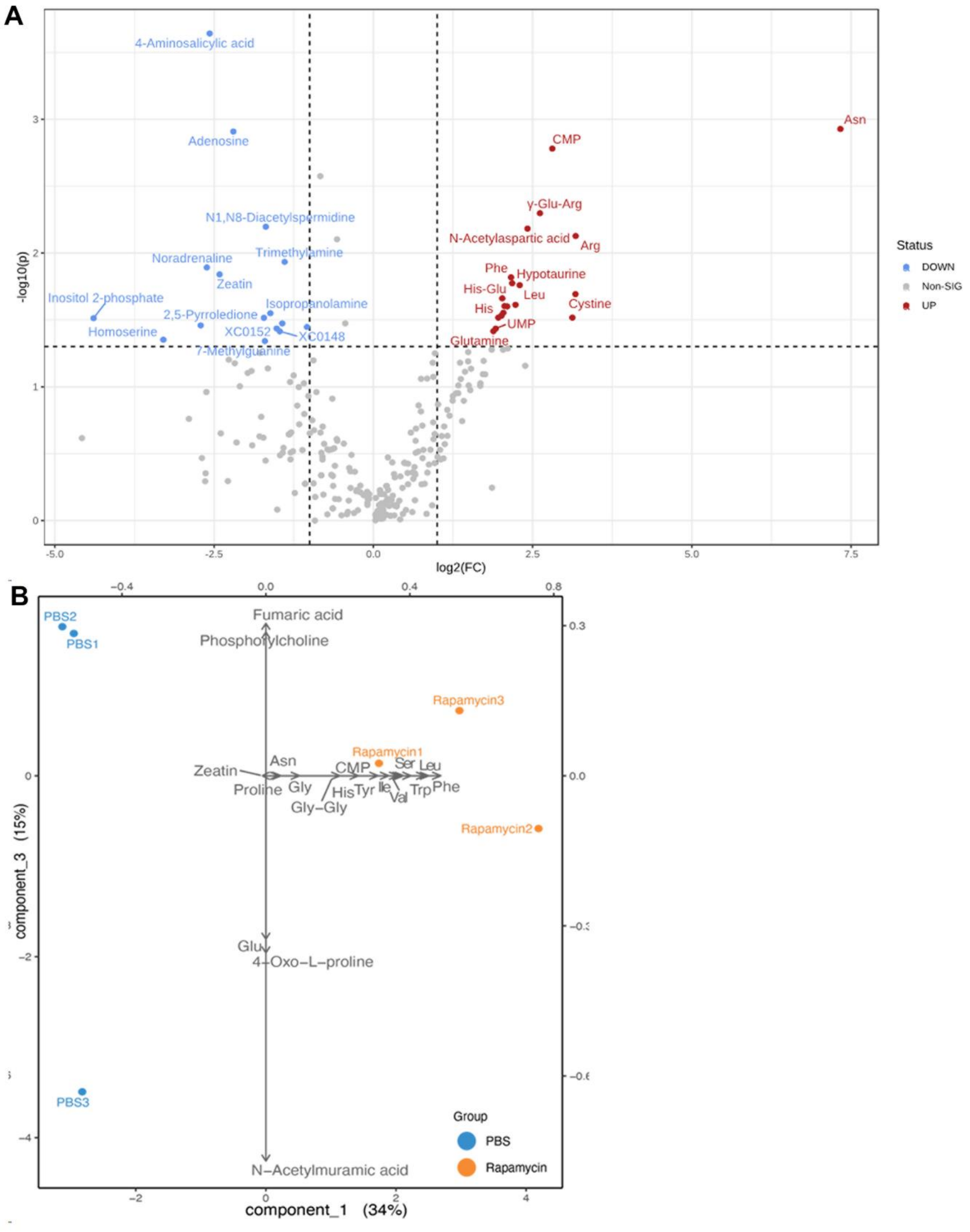

Supplemental Figure 8. Differentially abundant intraluminal metabolites under rapamycin treatment.

A) Volcano plot combines results from fold change (FC) analysis to show significantly increased

metabolites after rapamycin treatment for 7 days compared to no treatment control. Metabolites shown if FC is >2 and p value <0.05 based on 2-sample t-tests. Original metabolite measurement without normalization used in FC analysis. **B)** Biplot of intraluminal stool metabolome. Loading vectors and principal components labeled.

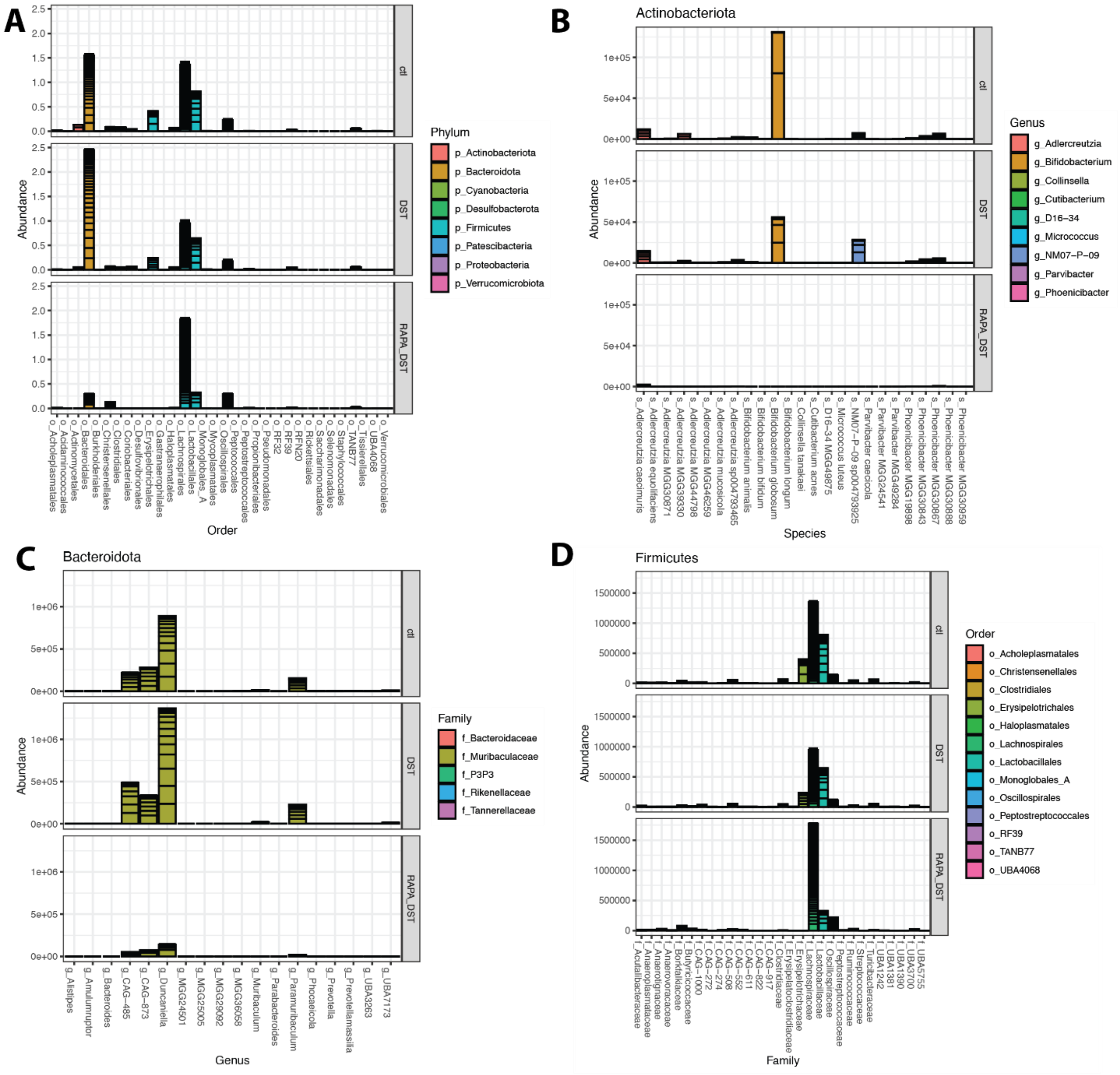

Supplemental Figure 9. Rapamycin influence on gut microbiome of alloantigen sensitized mice.

Comparison of relative abundance of bacterial groups in gut microbiota of alloimmune sensitized mice without and with rapamycin . Cumulative relative abundance of **A**) all taxonomic groups at order level, **B**) Actinobacteriota, **C**) Bacteroidota, and **D**) Firmicutes.

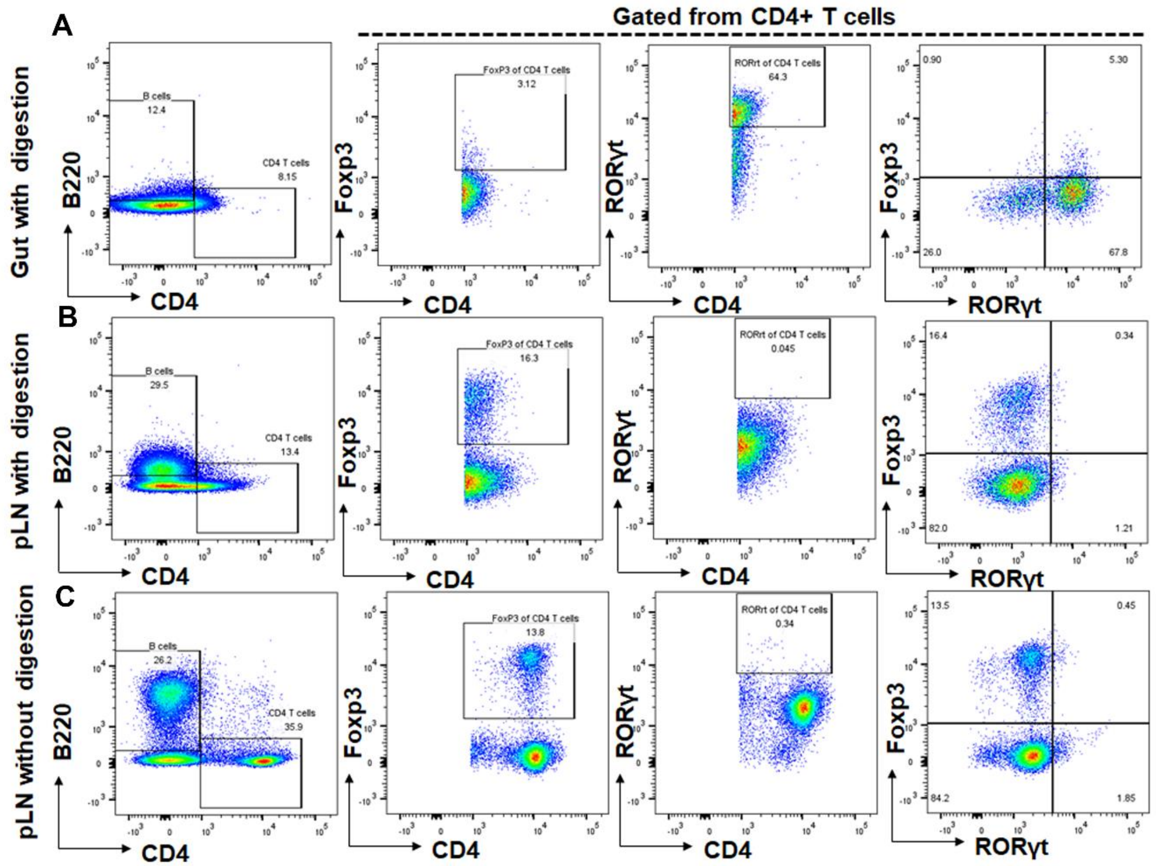

Supplemental Figure 10. Flow cytometric analysis of lymphocyte populations in gut and pLN.

Frequency of B220+ B cells (B220+), CD4+ T cells (CD4+), Foxp3+ Tregs (Foxp3+ CD4+), RORγt+ cells (RORγT+ CD4+) and RORγt+ Treg cells (Foxp3+ RORγt+ CD4+) in **A)** gut tissue digested with collagenase P, **B)** pLN processed with digestion, **C)** pLN processed without digestion.

Supplemental Tables

Supplemental Table 1. List of Antibodies

Target Molecule	Clone	Catalog No.
Anti-Hamster IgG Cy3	Polyclonal	Jackson ImmunoResearch; 127-165-160
Anti-Mouse IgG-AF647	Polyclonal	Jackson ImmunoResearch; 715-605-151
Anti-Rabbit AF488	Polyclonal	Jackson ImmunoResearch; 111-545-003
Anti-Rabbit Cy5	Polyclonal	Jackson ImmunoResearch; 111-175-003
Anti-Rabbit DL405	Polyclonal	Jackson ImmunoResearch; 711-476-152
Anti-Rabbit IgG-AF488	Polyclonal	Jackson ImmunoResearch; 711-545-152
Anti-Rabbit IgG-AF594	Polyclonal	Jackson ImmunoResearch; 111-585-003
Anti-Rabbit IgG-AF647	Polyclonal	Jackson ImmunoResearch; 711-606-152
Anti-Rat IgG AF594	Polyclonal	Jackson ImmunoResearch; 112-586-143
Anti-rat IgG AF647	Polyclonal	Jackson ImmunoResearch; 712-606-153
CD4	GK1.5	Biolegend; 100401
CD45	30-F11	Biolegend; 103132
CD31	390	Biolegend; 102427
CD8	53-6.7	Biolegend; 100701
ER-TR7	ER-TR7	Novus; NB100-64932
ER-TR7	Polyclonal	Santa Cruz; SC-73355
Foxp3	NRRF-30	eBioSci; 14477180
Foxp3	FJK-16s	eBioSci; 12-5773-82
Gp38	8.1.1	Biolegend; 127412
Laminin α4	775830	R&D; MAB3837
Laminin α5	Polyclonal	Novus Biol; NBP1-18714

Supplemental Table 2. Gut microbiome characteristics. A) Statistics of metagenomic sequencing of gut microbiome and time point. Fecal pellets obtained on days 3, 7, and 30. **B**) Gut microbiota taxonomic table characterized using the comprehensive mouse microbiota genome catalog (7). **c**) Microbial biomarkers using logarithmic linear discriminant analysis (LDA) effect size (LEfSe) (Segata et al., 2011). Alpha threshold value for pairwise non-parametric Kruskal-Wallis test was 0.05 and threshold for the logarithmic LDA model score for discriminative features was 2.0. All-against-all comparison in multi-class analysis performed.

Supplemental Table 3. Differential expressed genes (DEGs) between control and rapamycin group at **A**) day 7 and **B**) day 30. Significant differential expression assessed using DEseq2 (5) with an FDR value ≤ 0.05 and Fold-of-change (FC)>2. **C**) DEGs shared or unique to each time points. Result correspond to the UpSet plt in **Supplemental Figure 6A**.

Supplemental Table 4. Metabolome of intraluminal stool of no treatment control and rapamycin group after 7 days of treatment. **A**) Luminal metabolites; **B**) summarized by annotated functional pathways using KEGG BRITE hierarchical classification system (8).

Supplemental Table 5. Gut microbiome characteristics during allogeneic stimulation. A) Statistics of metagenomic sequencing of gut microbiome. **B**) Gut microbiota taxonomic table characterized using the comprehensive mouse microbiota genome catalog (7). **C**) Microbial biomarkers using logarithmic linear discriminant analysis (LDA) effect size (LEfSe) (Segata et al., 2011). Alpha threshold value for pairwise non-parametric Kruskal-Wallis test 0.05, and threshold for the logarithmic LDA model score for discriminative features 2.0. All-against-all comparison in multi-class analysis performed.

References:

1. M. Kanehisa, S. Goto, Y. Sato, M. Furumichi, M. Tanabe, KEGG for integration and interpretation of large-scale molecular data sets. *Nucleic Acids Res* **40**, D109-114 (2012).
2. E. A. Franzosa *et al.*, Species-level functional profiling of metagenomes and metatranscriptomes. *Nat Methods* **15**, 962-968 (2018).
3. R. Caspi *et al.*, The MetaCyc database of metabolic pathways and enzymes and the BioCyc collection of Pathway/Genome Databases. *Nucleic Acids Res* **42**, D459-471 (2014).
4. Y. Ye, T. G. Doak, A parsimony approach to biological pathway reconstruction/inference for genomes and metagenomes. *PLoS Comput Biol* **5**, e1000465 (2009).
5. S. Anders, W. Huber, Differential expression analysis for sequence count data. *Genome Biol* **11**, R106 (2010).
6. G. Yu, L. Wang, Y. Han, Q. He, clusterProfiler: an R package for comparing biological themes among gene clusters. *OMICS: A Journal of Integrative Biology* **16**, 284-287 (2012).
7. S. Kieser, E. M. Zdobnov, M. Trajkovski, Comprehensive mouse microbiota genome catalog reveals major difference to its human counterpart. *PLoS Comput Biol* **18**, e1009947 (2022).
8. M. Hattori, N. Tanaka, M. Kanehisa, S. Goto, SIMCOMP/SUBCOMP: chemical structure search servers for network analyses. *Nucleic Acids Res* **38**, W652-656 (2010).
